# Pyramid-level training with stain normalization for breast cancer lymph-node metastasis segmentation in whole-slide images

**DOI:** 10.1371/journal.pone.0362258

**Published:** 2026-11-13

**Authors:** Hamza Almobayed, Ali Yilmaz, Umit Senturk, Selma Erdogan Duzcu, Alperen Akgül

**Affiliations:** 1 Department of Computer Engineering, Bolu Abant İzzet Baysal University, Bolu, Turkey; 2 Department of Pathology, Bolu Abant Izzet Baysal University, Faculty of Medicine, Bolu, Turkey; Islamia University of Bahawalpur: The Islamia University of Bahawalpur Pakistan, PAKISTAN

## Abstract

Whole-slide histopathology images encode metastatic tissue information simultaneously at multiple spatial scales, from fine cellular morphology at high magnification to broad tissue architecture at low magnification. Existing segmentation models commonly operate at a single fixed resolution, leaving complementary information distributed across the whole-slide image pyramid underused. We present a multilevel segmentation framework with stain-normalization preprocessing that trains a unified model jointly across four study-defined WSI pyramid views (5×–40×), integrating Vahadane stain normalization and structured class-balanced sampling stratified by pyramid level, dataset, and tissue class. Applied to a 280-slide CAMELYON16/CAMELYON17 study cohort under strict slide-level data separation (196 training, 42 validation, 42 held-out test slides), the framework achieves a Dice coefficient of 92.39% on the held-out test set. Relative to the single-scale U-Net baseline (89.00%), the paired slide-level improvement is 3.39 percentage points (95% bootstrap CI: 3.19–3.60 pp; Cohen’s $d_{z}=4.98$; two-sided permutation *p* < 0.00001). Per-level evaluation confirms that full pyramid fusion outperforms each individual scale, while a boundary-focused L1 audit yields a mean HD95 of 53.88 pixels (95% CI: 50.28–57.64). The contribution is therefore a training-sampling and evaluation pipeline rather than a new network layer or architecture. These results show that multiresolution training, stain normalization, structured sampling, and leakage-controlled evaluation can materially improve breast lymph-node metastasis segmentation on established WSI benchmarks. A supplementary external cross-dataset evaluation on 103 paired BCSS breast-histopathology ROIs, performed without retraining or fine-tuning, yielded a pooled Dice of 70.58%, IoU of 54.54%, precision of 55.95%, and recall of 95.57%; the mean per-ROI Dice was 67.89% (95% bootstrap CI: 65.00–70.76%). These external results demonstrate substantial sensitivity to tumor-like tissue under a different image source and semantic annotation scheme, while also exposing the expected effect of annotation granularity and class-definition mismatch on overlap and boundary metrics.

## Introduction

According to WHO, breast cancer accounts for approximately 2.3 million new cases and 685,000 deaths annually, making it the most frequently diagnosed cancer among women [1,2]. Diagnosis relies on histopathological examination of tissue biopsies, the gold standard for cellular-level assessment [3,4]. A critical determinant of prognosis and adjuvant treatment selection is metastatic spread to the axillary sentinel lymph nodes, where small foci, particularly micrometastases (0.2–2 mm) and isolated tumor cell clusters, carry direct staging implications yet are morphologically subtle and prone to being overlooked during routine pathological review [4]. However, manual examination is time-consuming and subject to interobserver variability; in a study of 115 pathologists interpreting breast biopsy specimens, overall concordance with expert consensus diagnoses was 75.3% [5].

Digital pathology has transformed histological examination by enabling gigapixel-resolution whole-slide digitization, making computational analysis of tissue morphology at scale technically feasible [6]. Within this domain, deep learning has emerged as the dominant paradigm for automated biomedical image analysis [7], with encoder-decoder architectures such as U-Net demonstrating strong segmentation performance by preserving spatial resolution through skip connections between encoder and decoder stages [8]. These approaches have since been extended to histopathology, where they have shown utility for dense pixel-level segmentation [9] and automated tumor detection in lymph node tissue [10].

A central limitation is reliance on patch-level rather than whole-slide evaluation [11]. Patch-based validation can overestimate performance due to spatial redundancy and data leakage when patches from the same slide appear in both training and test splits [11,12]. This form of evaluation bias has been systematically documented across multiple medical imaging domains, including histopathology, where slide-level partitioning is essential for obtaining unbiased performance estimates [12,13]. Thus, strict slide-level separation and whole-slide reconstruction are prerequisites for reliable benchmarking [11,12].

Beyond evaluation protocol, three practical challenges constrain real-world segmentation performance. Differences in hematoxylin and eosin preparation protocols across clinical sites introduce substantial color variation that degrades model generalization, a challenge that normalization approaches such as Macenko spectral decomposition and Vahadane sparse nonnegative matrix factorization aim to address by transferring stain statistics to a common reference standard [3,14–17]. The multiscale organization of histopathological tissue presents a complementary difficulty: metastatic deposits range from isolated tumor cells identifiable only at high magnification to macrometastases spanning millimeters of tissue, motivating multiresolution frameworks that capture features at multiple spatial scales simultaneously [18]. Architectures such as HookNet, Hover-Net, and UNet++ incorporate this principle through multiscale feature aggregation within a single inference pass [19–21]. Finally, the overwhelming predominance of non-tumor tissue in whole-slide images creates severe class imbalance that can bias model training toward the majority class, a problem that structured balanced sampling strategies have been shown to partially mitigate [22,23].

Recent advances in vision transformers and pathology foundation models have introduced powerful alternatives for computational pathology [24–26]. Methods such as TransUNet and Swin-UNet use self-attention to capture long-range spatial dependencies, while large pretrained pathology encoders can provide transferable representations. The present study does not claim architectural superiority over these model families. Instead, it isolates a complementary question: how much performance can be gained by improving multiresolution training, stain handling, sampling, and leakage-controlled evaluation while holding the segmentation architecture conventional. A direct head-to-head benchmark against current foundation models is therefore outside the experimental scope of this study and is acknowledged as a limitation.

The present study addresses this gap with a reproducible, leakage-controlled WSI segmentation pipeline that integrates four components (multiresolution pyramid-level training across all four native WSI magnification levels, 5×–40×, Vahadane stain normalization, a 16-bucket structured class-balanced sampling strategy, and strict slide-level partitioning) and demonstrates that their systematic combination produces material performance gains without requiring a new model architecture. The primary contribution is this integration and its controlled evaluation within the present CAMELYON study. Within this integration, the 16-bucket structured sampling protocol is the most technically distinctive element: by stratifying training data simultaneously by WSI pyramid level, dataset origin, and tissue class, and applying morphological clustering within each stratum, it ensures that every magnification level receives balanced and morphologically diverse training signal, enabling the shared model to learn complementary features across the full spatial hierarchy of the WSI. Supporting contributions include normalized coordinate encoding to capture spatial tissue topology. Previously published methods are discussed separately as literature context rather than as models retrained in this study.

## Materials and methods

The methodological workflow is organized from study-cohort construction and leakage-controlled slide separation through patch preparation, feature engineering and data construction, model training, and whole-slide evaluation. All stages use a common slide-level partition so that patches originating from the same WSI remain in a single subset throughout the pipeline. Fig 1 provides an overview of this end-to-end workflow before the individual stages are described in detail.

**Fig 1:**
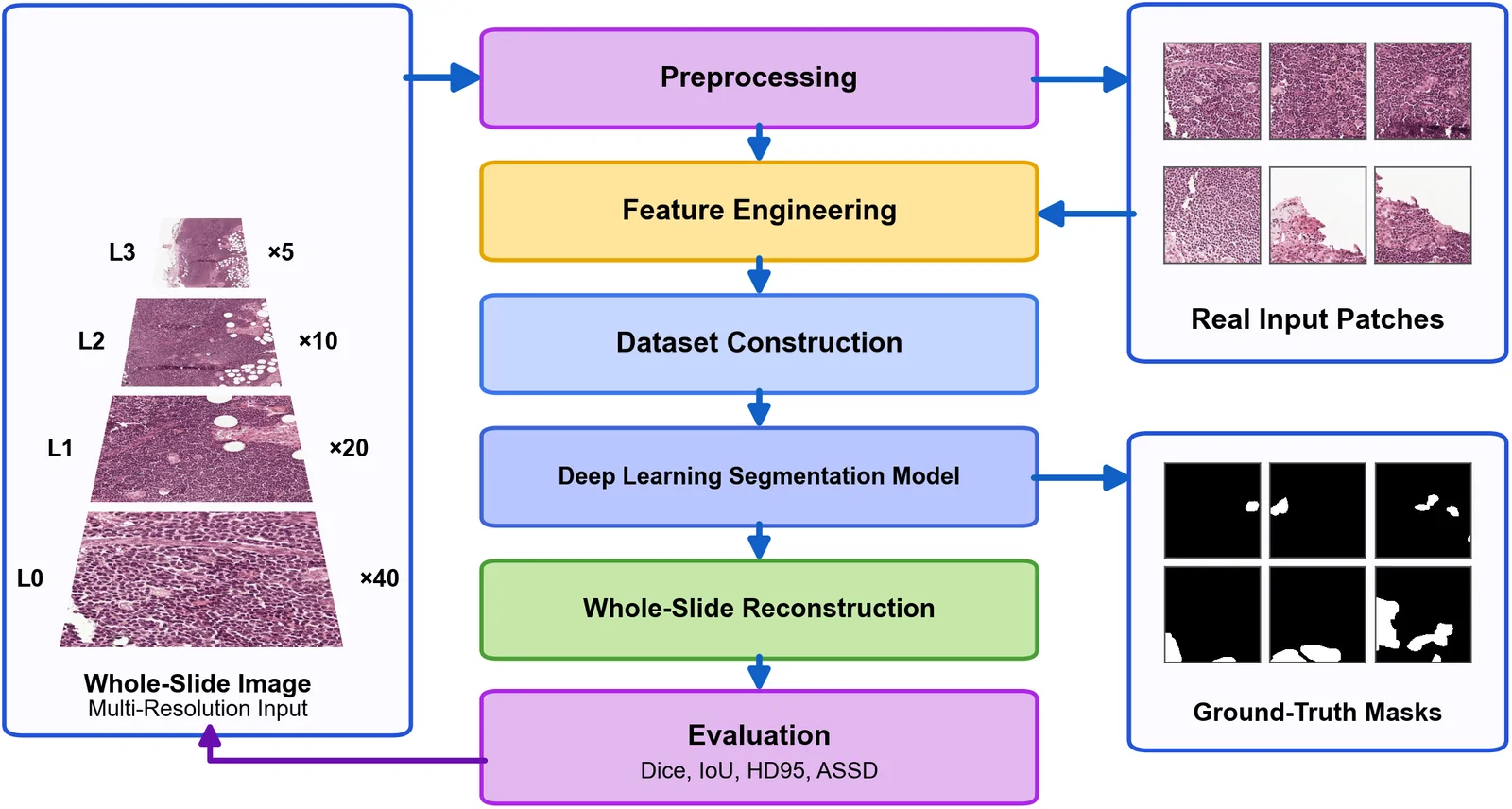
Overview of the complete pipeline. The input is a whole-slide image, which then passes through preprocessing and multiresolution patch extraction, feature engineering and dataset construction, model training, and whole-slide reconstruction. Evaluation is performed using segmentation and lesion-level metrics on the held-out test data.

### Dataset and slide-level data separation

This study evaluates a multilevel segmentation pipeline with stain-normalization preprocessing using the public CAMELYON16 and CAMELYON17 resources [27,28]. The official CAMELYON16 training set contains 270 WSIs from Radboud University Medical Center (RUMC) and University Medical Center Utrecht (UMCU), comprising 160 normal slides and 110 slides with metastases. CAMELYON17 extends the benchmark to 1,000 WSIs from 200 patients across five centers (RUMC, UMCU, Canisius-Wilhelmina Hospital [CWZ], Rijnstate Hospital [RST], and the Laboratory of Pathology East Netherlands [LPON]); detailed lesion-level annotations are available for 50 CAMELYON17 training WSIs (10 per center) [27,28]. These official benchmark counts are distinct from the study-specific cohort used in the experiments below.

The study-specific analysis pool comprised 280 WSIs: 120 from CAMELYON16 and 160 from CAMELYON17. Tumor-positive WSIs used the official CAMELYON lesion annotations, while negative WSIs were represented by all-zero masks; no manual tumor contours were created by the authors. The 280 slides were divided before patch sampling into 196 training, 42 validation, and 42 held-out test slides using a 70/15/15 slide-level partition. Specifically, CAMELYON16 contributed 84 training, 18 validation, and 18 test slides (120 total), while CAMELYON17 contributed 112 training, 24 validation, and 24 test slides (160 total). All patches derived from a WSI inherit that WSI’s partition. Because CAMELYON17 can contain multiple lymph-node WSIs from the same patient, patient identifiers were additionally audited when constructing the split to avoid cross-subset patient overlap. This separation prevents patch-level leakage across training, validation, and test sets [11–13].

In the final study protocol, a patch is assigned to the tumor class when annotated tumor occupies at least 10% of the corresponding ground-truth crop; patches below this threshold are assigned to the non-tumor class to reduce boundary-label noise from very small annotation overlap. Class-specific tissue and quality filters are then applied before sampling, and the subsequent 16-bucket selection stage enforces controlled class balance. The official CAMELYON masks are therefore the sole source of tumor supervision.

### Preprocessing and patch extraction

Preprocessing converts each WSI and its official CAMELYON annotation into quality-controlled, stain-normalized multiresolution patches suitable for model training. The sequence includes tissue masking, exclusion of blank/background regions, Cartesian patch extraction, quality filtering, XML annotation rasterization, tumor/normal label assignment, and stain normalization. The complete preparation sequence is summarized in Fig 2.

**Fig 2:**
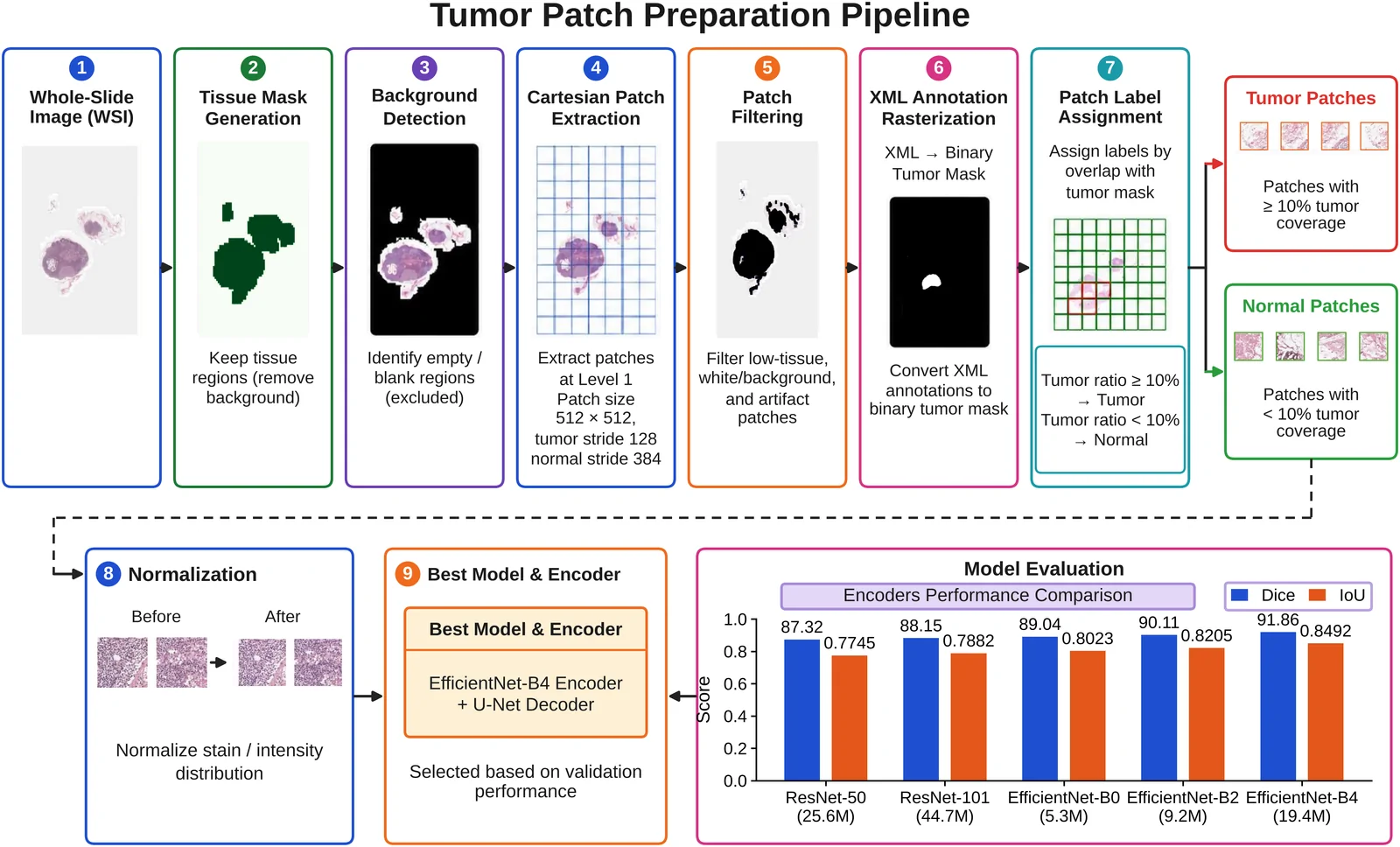
Tumor patch preparation pipeline. Whole-slide images are restricted to tissue regions, blank/background areas are excluded, and 512×512 patches are extracted on the reference grid. Low-quality patches are filtered, official XML annotations are rasterized into binary tumor masks, and retained patches are labeled according to tumor coverage before stain normalization and downstream model selection.

Tissue regions are identified from a low-resolution WSI view using the TIAToolbox morphological tissue masker, with small isolated regions removed before patch selection. Patches of 512×512 pixels are then stored from four study-defined pyramid views corresponding approximately to L1 (40×), L2 (20×), L3 (10×), and L4 (5×) effective magnification [18,29]. Tumor candidate regions are sampled densely using a 128-pixel stride, whereas normal candidate regions are sampled more sparsely using a 384-pixel stride to reduce redundant background sampling. Candidate selection is performed in a common reference coordinate system so that corresponding spatial locations can be saved across the requested pyramid views. Higher magnification captures fine cellular morphology, while lower magnification encodes broader tissue architecture, together supporting detection of both small and large metastatic deposits [19,21], as illustrated in Fig 3.

**Fig 3:**
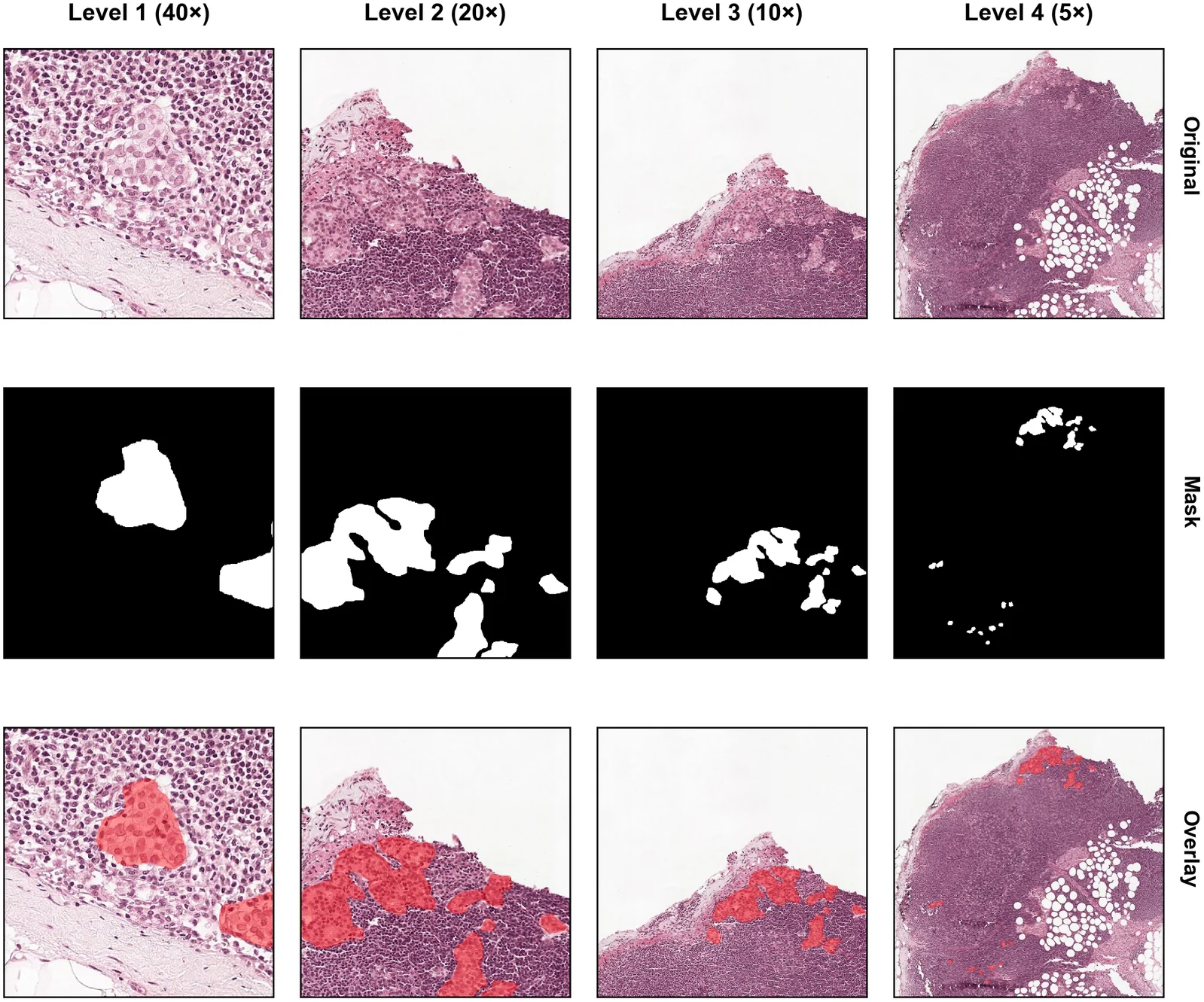
Multiresolution patch extraction from the same tissue region at four magnification levels. The left side shows patches extracted at Level 1 (×40), Level 2 (×20), Level 3 (×10), and Level 4 (×5), with decreasing detail from top to bottom. The right side displays, for the same region, the original image, the ground truth mask, and the overlay of the mask on the original image.

Patch quality control is class-aware. Normal candidates require at least 70% tissue coverage, a white-pixel ratio no greater than 0.55, sufficient purple H&E content, a maximum dark-pixel fraction of 0.25, and Laplacian variance of at least 35. Tumor candidates use a less restrictive tissue threshold (20%), white-pixel ratio no greater than 0.85, maximum dark-pixel fraction of 0.60, and Laplacian variance of at least 15 so that small or pale metastatic regions are not preferentially discarded. These thresholds follow the extraction implementation used for the stored patch set. Each retained patch is then normalized using Vahadane stain normalization, which applies sparse nonnegative matrix factorization to separate hematoxylin and eosin stain components and transfer them to a common reference standard [16]. The reference slide was selected from CAMELYON16 training data based on visual inspection for representative H&E quality, characterized by well-defined eosin background, clear nuclear hematoxylin staining, and absence of visible artifacts. All patches from both CAMELYON16 and CAMELYON17 are normalized to match the stain density statistics of this reference, reducing cross-center color variation without altering tissue morphology (Fig 4). This approach has been shown to outperform Macenko spectral decomposition and unnormalized baselines in cross-slide consistency [15–17].

**Fig 4:**
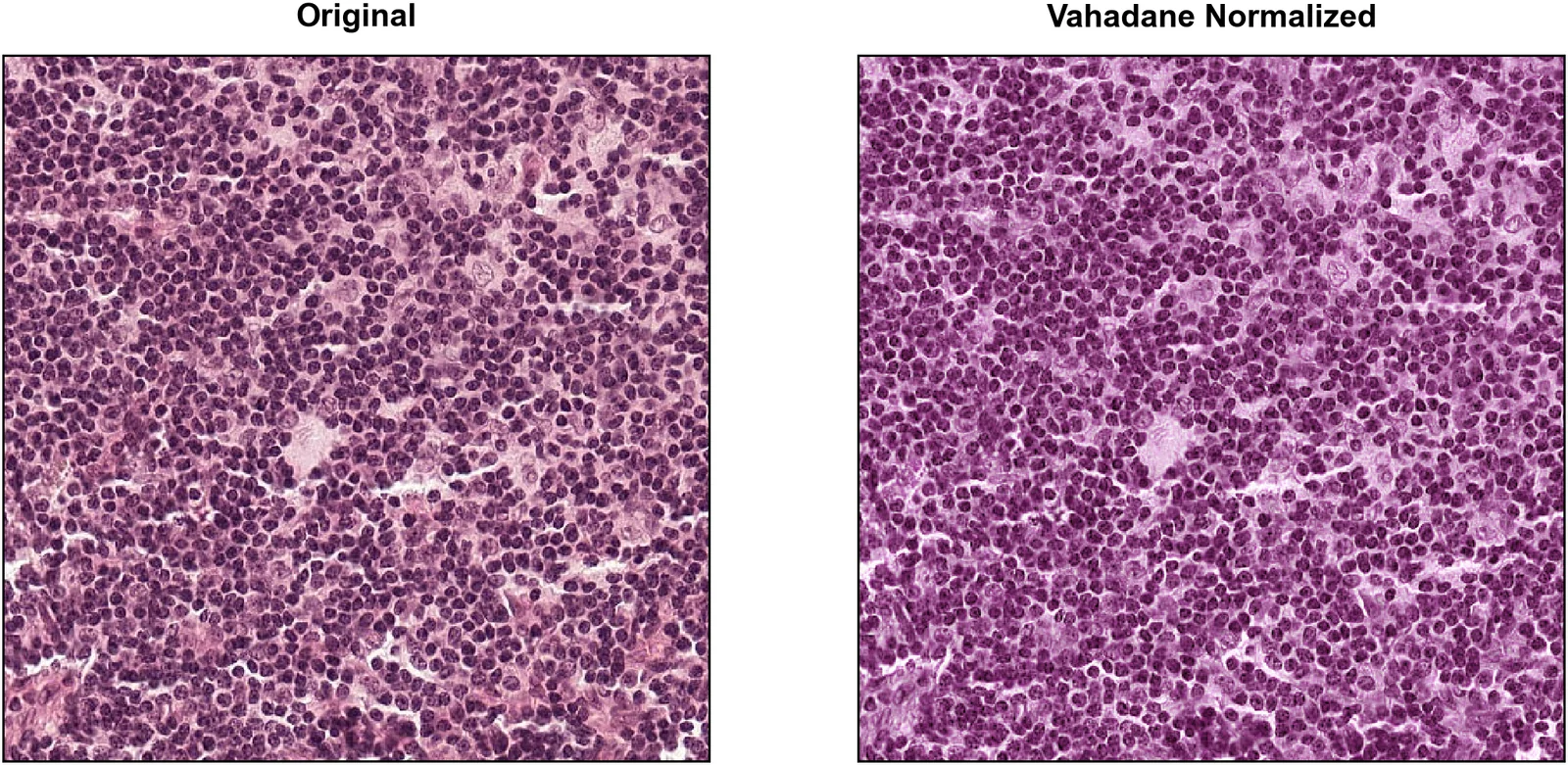
Stain normalization using the Vahadane method. The left image shows the original patch before normalization. The right image shows the same patch after Vahadane normalization, with reduced stain variability across different slides.

### Feature engineering and data construction

After preprocessing and stain normalization, retained patches are converted into a structured analysis pool designed to preserve morphology while controlling scale, source, and class imbalance. The construction stage operates independently within the 16 combinations of dataset source, pyramid level, and tissue class, and then passes the resulting slide-linked samples to the fixed slide-level train/validation/test partition.

For structured sampling, EfficientNet-B4 features are extracted from each retained patch, producing 1,792-dimensional vectors that encode nuclear density, texture, and glandular architecture [30]. Because naive random sampling from WSIs produces severe class imbalance due to the predominance of non-tumor tissue, each patch is assigned to one of 16 buckets defined by a unique combination of dataset (2 options), magnification level (4 levels), and class label (2 labels), so that each bucket contains patches of a single class from a specific scale and dataset, as depicted in Fig 5. Within each bucket, EfficientNet-B4 feature vectors are clustered by K-means, with the number of clusters selected independently for that bucket using an elbow criterion up to a maximum of 12 clusters (random seed 42). Patches are then sampled across the resulting clusters to reach approximately 5,000 patches per bucket, preserving morphological diversity while enforcing the planned 16-bucket balance [22,23,31]. This yields a balanced 80,000-patch analysis pool (approximately 5,000 samples per bucket; 40,000 tumor and 40,000 non-tumor patches) from which the slide-linked training, validation, and test subsets are used according to the predefined slide partition, without assuming a single fixed *K* for all buckets.

**Fig 5:**
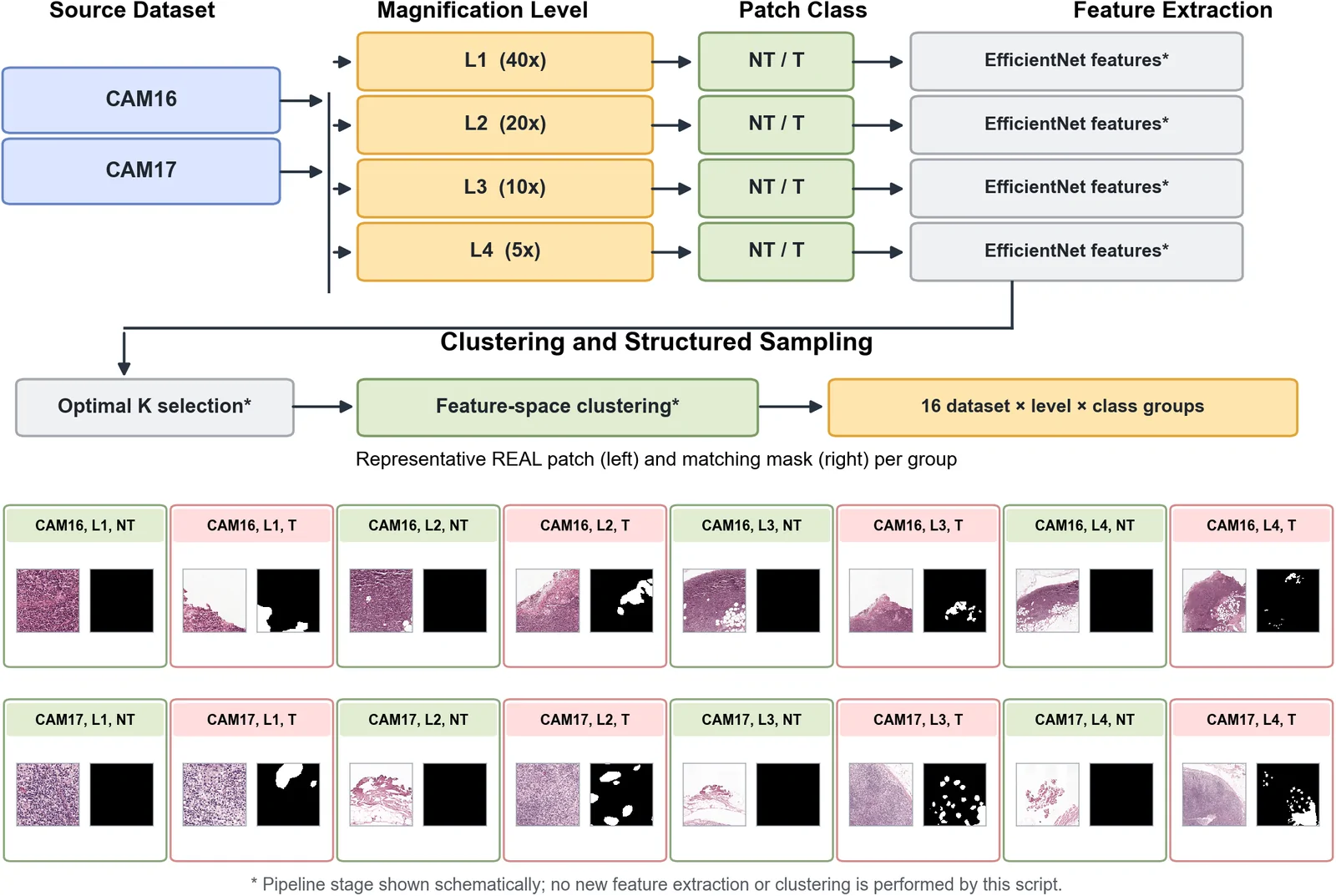
Structured sampling with clustering. The figure details the sixteen-bucket sampling strategy. Source datasets include CAMELYON16 and CAMELYON17. Four magnification levels are considered: Level 1 (×40), Level 2 (×20), Level 3 (×10), and Level 4 (×5). Each dataset, magnification level, and label (Tumor or Non-Tumor) defines a distinct bucket. For every bucket, EfficientNet feature vectors are extracted, and K-means clustering determines the optimal number of clusters. Subsequently, a fixed number of patches are sampled per bucket, yielding a total of sixteen distinct buckets for training.

The resulting 16-bucket feature-engineering and sampling design is summarized in Fig 5.

A strong augmentation pipeline is applied to training patches [32] (Fig 6): horizontal and vertical flips (p  =  0.5 each), random rotations in 90° increments, color jitter (brightness ±0.25, contrast ±0.25), random gamma (γ∈[0.7,1.5]), Gaussian blur (kernel 3×3), additive Gaussian noise (σ≤12.0), and JPEG compression (quality ≥35). All augmentations are applied only to training patches. Validation and test patches receive only Vahadane normalization.

**Fig 6:**
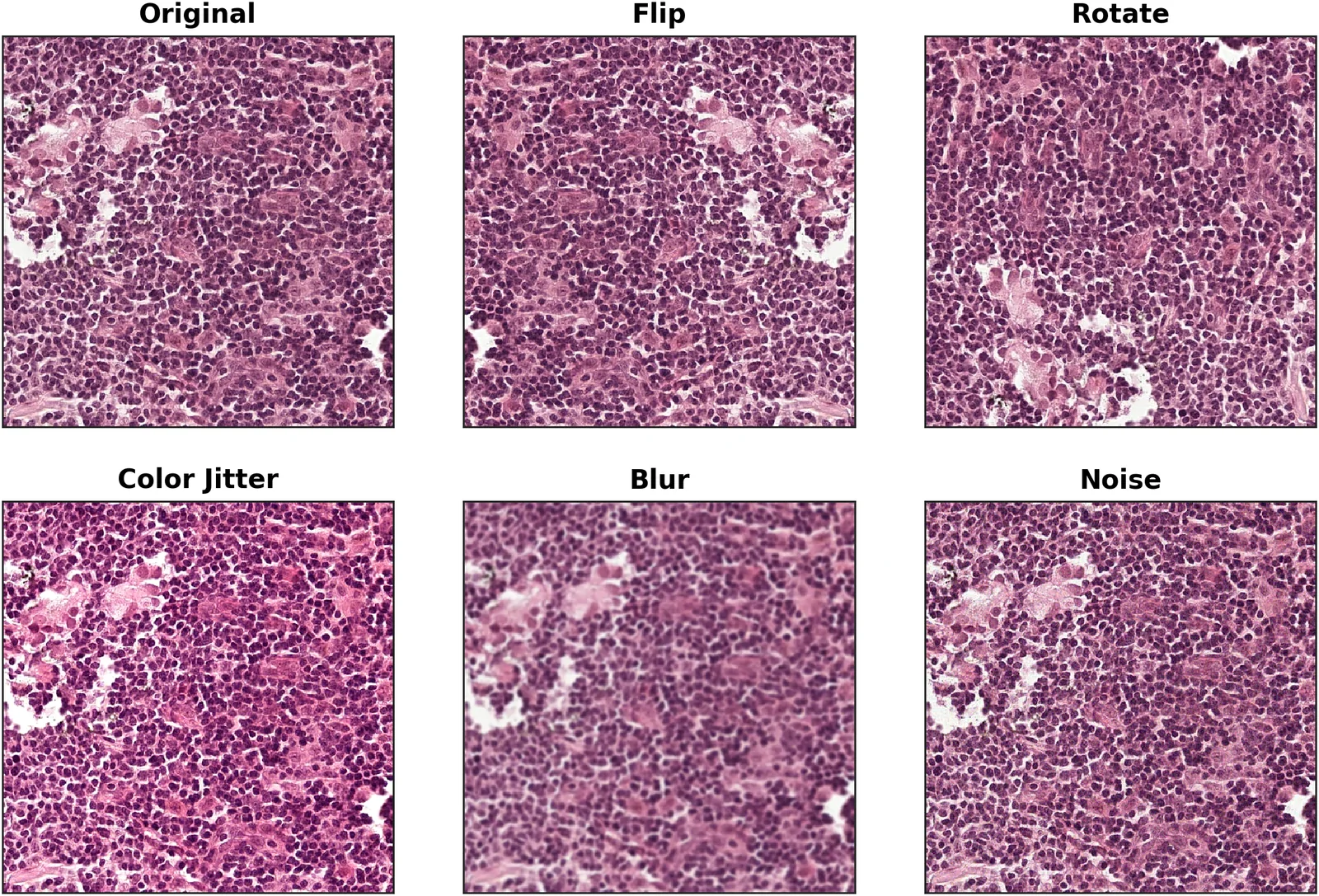
Training augmentation visualization. The figure displays the original image alongside five commonly used augmentation techniques: flip, rotation, color jitter, Gaussian blur, and random noise. These augmentations are applied during training to increase the effective dataset size, reduce overfitting, and enhance model generalization to unseen histopathology images.

### Segmentation architecture and training

All experiments were implemented in Python 3.10 using PyTorch and OpenSlide/TIAToolbox-based WSI access. As shown in Fig 7, the model uses a U-Net-style decoder with an ImageNet-pretrained EfficientNet-B4 encoder [8,30], preserving multiscale spatial information through skip connections [8,21]. Patch size is fixed at 512×512 pixels. Patch extraction uses the class-dependent strides described above (128 pixels for tumor candidates and 384 pixels for normal candidates); there is no universal 256-pixel extraction stride in the final data pipeline.

**Fig 7:**
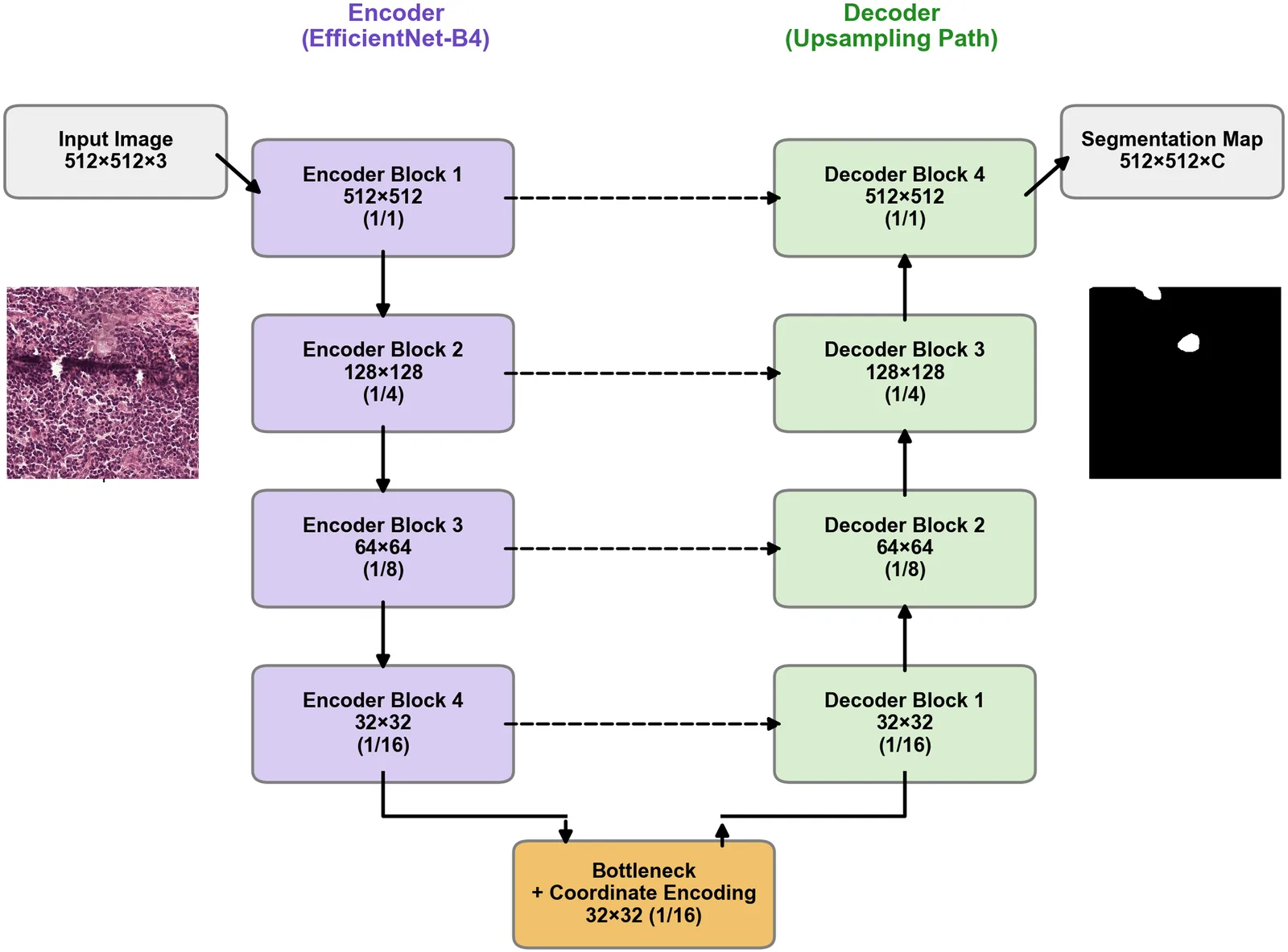
U-Net architecture with EfficientNet-B4 encoder. The encoder takes an input image and progressively downsamples feature maps through five encoder blocks at decreasing resolutions. The bottleneck block includes coordinate encoding to preserve spatial information. The decoder upsamples feature maps through five decoder blocks, with skip connections from the corresponding encoder blocks. The final output is a segmentation map.

To select the optimal encoder, several pretrained backbones were evaluated under identical training conditions (single-resolution at L2, 20×, no stain normalization, random sampling) on the validation set, including ResNet-50 and ResNet-101 [33] as well as EfficientNet-B0, B2, and B4 [30]. Under this controlled comparison (Table 1), EfficientNet-B4 (19.4 M parameters) achieves the highest validation Dice of 91.86% and IoU of 0.8492, exceeding ResNet-50 (87.32%, 25.6 M) by 4.54 percentage points despite using approximately 24% fewer parameters, and improving on the smaller EfficientNet-B0 (89.04%, 5.3 M) by 2.82 percentage points. ResNet-101, the largest model evaluated (44.7 M parameters), reaches only 88.15% Dice. In this setting, parameter count alone does not predict segmentation accuracy, and the EfficientNet compound-scaling design offers a more favorable accuracy-to-parameter tradeoff. EfficientNet-B4 is therefore retained as the encoder in all subsequent experiments.

**Table 1:** Comparison of encoder architectures.

Encoder	Parameters (M)	Dice (%)	IoU
ResNet-50	25.6	87.32	0.7745
ResNet-101	44.7	88.15	0.7882
EfficientNet-B0	5.3	89.04	0.8023
EfficientNet-B2	9.2	90.11	0.8205
**EfficientNet-B4**	19.4	**91.86**	**0.8492**

All encoders were trained under identical conditions (single-resolution at L2, 20×, no stain normalization, random sampling) and evaluated at the patch level. EfficientNet-B4 achieved the highest patch-level Dice (91.86%) and IoU (0.8492) with 19.4M parameters. The baseline single-resolution model was trained at L2 (20×) magnification. Patch-level evaluation produces higher absolute scores than slide-reconstruction evaluation on the same model. The primary single-scale baseline used the same EfficientNet-B4 U-Net backbone as the proposed framework but was restricted to L2, random sampling, and no stain normalization; its ablation/validation performance and held-out reconstructed test performance are reported in their respective tables, so those values should not be conflated with literature U-Net results in Table 12.

Normalized patch coordinates (the patch center position within the WSI, scaled to [0,1] along both axes) are processed through a two-layer MLP (input: 2 → hidden: 64 → output: 32, with ReLU activations between layers) and concatenated channel-wise with the bottleneck feature map of the encoder before the first decoder upsampling block. This encoding allows the model to learn spatially varying tissue appearance patterns: subcapsular sinus regions, cortical follicles, and medullary areas exhibit distinct morphological signatures at different spatial positions within a lymph node cross-section. This spatial context helps the decoder account for region-specific morphological patterns. Ablation studies confirm that coordinate encoding improves Dice over the same configuration without positional input (Table 2). To verify that this gain reflects genuine anatomical learning rather than incidental regularization, a control experiment was performed by shuffling the coordinates within each validation slide (random permutation of patch coordinates while preserving the set of patches). Under this shuffled-coordinate control, the validation Dice dropped from 95.12% to 94.65% (Δ=0.47 pp), confirming that the majority of the gain (0.47 of 0.74 pp, approximately 64%) is attributable to spatial anatomy learning, while the remaining 0.27 pp reflects regularization effects. The decoder comprises four upsampling blocks, each containing bilinear upsampling followed by two convolutional layers with ReLU activations and skip connections from the corresponding encoder stage. Dropout (rate 0.1) is applied after each block. The final 1×1 convolution produces a scalar logit map.

**Table 2:** Ablation study on the validation set.

Configuration	Dice (%)	IoU	Precision	Recall
*Incremental component build-up*				
Baseline (single-res, random sampling, no norm.)	88.46	0.7934	0.9742	0.8147
+ Vahadane stain normalization	90.28	0.8231	0.9806	0.8367
+ Multiresolution extraction (4 levels)	92.15	0.8549	0.9854	0.8572
+ Class-balanced sampling (no K-means)	93.12	0.8745	0.9878	0.8710
+ 16-bucket balanced sampling (with K-means)	94.38	0.8934	0.9903	0.8829
+ Coordinate encoding (**full pipeline**)	**95.12**	**0.9069**	**0.9918**	**0.8915**
*Stratification check (both configurations slide-disjoint)*				
Full pipeline, unstratified random slide-level split	89.67	0.8124	0.9778	0.8281

Starting from a baseline configuration (single-resolution at L2, 20×, random sampling, no normalization), components are added sequentially. Paired slide-level Dice statistics for the incremental comparisons were: Vahadane vs. baseline +1.82 pp (95% CI 1.72–1.93; $d_{z}=5.14$), multiresolution vs. Vahadane +1.87 pp (1.81–1.93; $d_{z}=9.38$), class-balanced vs. multiresolution +0.97 pp (0.91–1.03; $d_{z}=4.94$), K-means structured sampling vs. class-balanced +1.26 pp (1.20–1.32; $d_{z}=6.00$), coordinate encoding vs. K-means +0.74 pp (0.67–0.80; $d_{z}=3.50$), and full stratified pipeline vs. unstratified split +5.45 pp (5.35–5.55; $d_{z}=16.48$). All remained significant after Holm correction ($p_{Holm}<0.001$). The order of addition influences the measured gain of each component, so the increments are specific to this build-up sequence.

The base training hyperparameters are: per-GPU batch size of 8, initial learning rate $1\times 10^{-4}$, weight decay $1\times 10^{-4}$, linear warm-up over 3 epochs, and a maximum of 50 epochs (with early stopping as described below). All reported configurations use these same hyperparameter values to ensure comparability.

Training uses AdamW [34] with learning rate $1\times 10^{-4}$ and weight decay $1\times 10^{-4}$. The final segmentation objective is BCEWithLogitsLoss; a Dice term is not included in the final loss implementation. Automatic mixed precision (FP16) is enabled to reduce memory consumption [35]. The per-GPU batch size is 8 with gradient accumulation over 8 steps (effective batch size 64), and gradient clipping is applied at a norm of 1.0. Early stopping monitors validation Dice with a patience of 7 epochs and restores the best checkpoint. The maximum number of epochs is 50; the reported training run selected the best checkpoint at epoch 28 and stopped at epoch 35. Training was conducted on a single NVIDIA GeForce RTX 4050 GPU (6 GB VRAM, 16 GB system RAM).

This complete configuration (EfficientNet-B4 encoder, four-level multiresolution extraction, 16-bucket structured sampling, Vahadane stain normalization, and normalized coordinate encoding) constitutes the full pipeline used in all subsequent experiments [16,30]. Each component was selected from the per-component ablation choices that gave the highest validation Dice under the controlled retraining protocol, and the same configuration is held fixed across the test-set evaluation, per-magnification analysis, per-center evaluation, leave-one-center-out cross-validation, and the lesion-level FROC analysis reported in the Results section. Holding the architectural and data-handling configuration constant across all reported experiments ensures that observed performance differences between configurations, magnification levels, and centers reflect data-level design choices rather than confounds from incidental hyperparameter changes.

### Whole-slide inference and reconstruction

For whole-slide evaluation, patch probability maps are returned to their recorded WSI coordinates and combined in WSI space. Where patch predictions overlap, probabilities are averaged before binarization. Per-level probability maps are reconstructed separately and resampled to the common L1 evaluation frame before pixel-wise four-level averaging. Regions outside the tissue mask are excluded from the reconstructed evaluation map. The final fused probability map is binarized at a fixed probability threshold of 0.50, matching the final training and evaluation code. No test-set threshold optimization is performed.

It is important to distinguish reconstructed WSI evaluation from the sampled diagnostic analyses. The approximately 1.67 billion pixel positions reported for the broader patch-based audit refer to aggregate pixel positions processed across sampled 512×512 patches and are not the number of unique native WSI pixels; overlapping windows can count the same tissue location more than once. Separately, the confusion-matrix visualization in Fig 10 is a compact 12,000-patch diagnostic summary (3,000 patches per level) and should not be interpreted as the source of the 1.67-billion-pixel audit count or as a deployment-prevalence estimate.

### Performance metrics

Primary segmentation metrics are Dice similarity coefficient [36] and intersection over union (IoU/Jaccard), with precision and recall reported to characterize the false-positive/false-negative trade-off. Pixel accuracy is reported only as a secondary descriptive metric because of the strong class imbalance in WSI tissue. Binary masks are obtained using a fixed threshold of 0.50, consistent with the final evaluation implementation.


$$\begin{array}{c} \text{Dice}=\frac{2\times TP}{2\times TP+FP+FN},\;\text{IoU}=\frac{TP}{TP+FP+FN} \end{array}$$ (1)

$$\begin{array}{c} \text{Precision}=\frac{TP}{TP+FP},\;\text{Recall}=\frac{TP}{TP+FN},\;\text{Accuracy}=\frac{TP+TN}{TP+TN+FP+FN} \end{array}$$ (2)

For the primary proposed-versus-baseline comparison and other analyses for which paired slide-level values were available, inference was performed at the slide level. Mean paired differences and 95% confidence intervals were estimated by paired bootstrap resampling with 20,000 resamples [37]. Two-sided statistical significance was assessed using paired sign-flip permutation tests with 200,000 Monte Carlo permutations [38]. Effect size is reported as paired-sample Cohen’s $d_{z}$. To control family-wise error across related secondary comparisons, Holm’s sequentially rejective procedure was applied separately to the ablation, stain-normalization, and pyramid-level comparison families [39]. Comparisons for which only aggregate summary values were available are retained as descriptive rather than assigning unsupported *p*-values.

### Boundary-focused evaluation

Because overlap metrics alone can conceal clinically relevant contour errors, a complementary post-hoc boundary analysis was performed on 2,694 held-out L1 patches using the 95th-percentile Hausdorff distance (HD95) in pixels [40]. Among patches for which a valid tumor boundary distance could be computed, the mean HD95 was 53.88 pixels, the median was 22.0 pixels, and the bootstrap 95% CI for the mean was 50.28–57.64 pixels. The corresponding pooled Dice in this boundary-audit subset was 0.9375. HD95 is reported in pixels because the analysis was performed directly in the L1 evaluation grid and is intended as a complementary boundary-error measure rather than a replacement for the primary WSI Dice analysis.

### External cross-dataset validation on BCSS

To complement the CAMELYON in-domain and leave-one-center-out analyses with an independent public image source, the final CAMELYON-trained model was evaluated without retraining or fine-tuning on the Breast Cancer Semantic Segmentation (BCSS) dataset described by Amgad et al. [41]. BCSS contains breast-cancer histopathology regions of interest derived from TCGA and provides indexed pixel-wise semantic tissue labels at 0.25 μ m/pixel. The external evaluation used the same inference configuration as the primary model: 512×512 patches, 128-pixel sliding stride, Vahadane normalization, and a fixed probability threshold of 0.50. BCSS label 1 was treated as tumor; labels 0 (outside the annotated ROI) and 7 (exclude) were omitted from scoring, and the remaining valid tissue labels were treated as non-tumor. Overlapping patch probabilities were averaged to reconstruct each ROI before thresholding. Dice and HD95 were computed against the BCSS tumor reference mask on valid pixels only. After automated scoring, an experienced pathologist qualitatively reviewed representative discordant BCSS ROIs in which model predictions extended beyond the class-1 reference mask. This expert review was used only to interpret selected discrepancies; it did not alter the BCSS ground truth, re-label the dataset, or change any quantitative metric.

This external test is intentionally interpreted as cross-dataset breast-histopathology validation rather than as a direct replication of the CAMELYON lymph-node task. CAMELYON16/17 evaluate metastatic breast-carcinoma deposits within lymph-node tissue, whereas BCSS represents primary breast-cancer histopathology regions in breast-tissue specimens. This anatomical and histological context shift changes the surrounding tissue architecture and tumor microenvironment in addition to the semantic labeling protocol and annotation granularity. Consequently, disagreement between a predicted tumor-like region and the BCSS class-1 mask is counted as a false positive under strict pixel-wise scoring even when the region is morphologically plausible. Representative discrepant cases were subsequently reviewed qualitatively by a pathologist, who judged that some predicted areas extending beyond the original class-1 mask contained tumor on histopathologic morphology. These expert observations were used only for interpretation; the original BCSS masks remained unchanged for all quantitative scoring. This distinction is important when interpreting Dice and boundary metrics under imperfect or heterogeneous reference annotations [42,43].

### Ethics statement

This study uses only publicly available, fully de-identified CAMELYON16, CAMELYON17, and BCSS histopathology datasets released for open scientific use. No new patient data were collected and no participants were prospectively recruited. Accordingly, no additional institutional ethics approval was required for this computational analysis.

## Results

The proposed framework was evaluated on a held-out test set of 42 slides (18 from CAMELYON16 and 24 from CAMELYON17), strictly separated from all training and validation data at the slide level. The subsections below report the incremental contribution of each pipeline component through ablation, comparisons across stain normalization strategies, final whole-slide segmentation performance with bootstrap confidence intervals, per-magnification and cross-center breakdowns, and a structured analysis of the principal failure modes.

### Ablation study

To quantify the individual contribution of each pipeline component, Table 2 presents an incremental ablation evaluated on the validation set under an identical 70/15/15 slide-level split and training protocol. All configurations share the same EfficientNet-B4 backbone, so performance differences reflect data-level design choices rather than architectural variation. To assess the stability of the 70/15/15 split, a 5-fold cross-validation was performed on the training and validation sets (196 + 42 = 238 slides). The mean validation Dice across five folds was 94.87% (SD = 0.42%), ranging from 94.32% to 95.41%, confirming that the reported 95.12% validation Dice is representative and not an artifact of a particular split composition. The 5.45 percentage point gap between the stratified slide-level split (95.12%) and the unstratified random split (89.67%) highlights the sensitivity of reported performance to split composition and underscores the importance of stratified partitioning for stable benchmarking.

Each added component produces a monotone increase in validation Dice. The single-resolution random-sampling baseline reaches 88.46%. Vahadane stain normalization raises Dice by 1.82 percentage points to 90.28%. Multiresolution extraction across the four pyramid levels contributes one of the largest gains in the pipeline (90.28%→92.15%, + 1.87 pp), which shows the direct benefit of training jointly across the full WSI pyramid rather than at a fixed magnification. Standard class-balanced oversampling adds a further 0.97 pp (to 93.12%), and replacing it with the 16-bucket K-means morphological clustering scheme adds another 1.26 pp (to 94.38%). The 1.26 pp gap between these two rows confirms that morphological diversity within each bucket is independently beneficial beyond simple class balance. Coordinate encoding contributes a final 0.74 pp (94.38%→95.12%) and completes the full pipeline. The stratification check row compares this full pipeline under the stratified slide-level partition (95.12%) with an unstratified random slide-level split (89.67%). Both configurations are slide-disjoint, so neither condition contains patch-level data leakage. Nevertheless, the unstratified split yields a 5.45 percentage point drop in validation Dice. This gap shows that imbalance in class and acquisition-center representation across train and test partitions is a first-order determinant of reported accuracy and motivates stratified slide-level partitioning for stable benchmarking.

### Stain normalization strategy comparison

To isolate the contribution of Vahadane normalization relative to alternative stain-handling strategies, Table 3 compares four conditions under the single-resolution, random-sampling baseline configuration. All other training settings are held constant.

**Table 3:** Comparison of stain normalization strategies on the validation set.

Stain Strategy	Dice (%)	IoU	Precision	Recall
No normalization	88.46	0.7934	0.9742	0.8147
Macenko normalization	89.12	0.8036	0.9765	0.8218
Stain augmentation only	87.94	0.7852	0.9721	0.8063
**Vahadane normalization**	**90.28**	**0.8231**	**0.9806**	**0.8367**

All strategies were evaluated using a single-resolution (L2, 20×) baseline with random sampling. Relative to Vahadane, the paired Dice differences were +1.82 pp vs. no normalization (95% CI 1.72–1.93; $d_{z}=5.14$), + 1.16 pp vs. Macenko (0.96–1.37; $d_{z}=1.71$), and +2.34 pp vs. stain augmentation (2.16–2.54; $d_{z}=3.65$). All three comparisons remained significant after Holm correction ($p_{Holm}<0.001$).

Vahadane normalization yields the highest validation Dice of 90.28% with IoU 0.8231, outperforming Macenko (89.12%, IoU 0.8036) by 1.16 percentage points and the unnormalized baseline (88.46%, IoU 0.7934) by 1.82 percentage points. Stain augmentation alone reaches only 87.94% Dice (IoU 0.7852), falling 0.52 percentage points below the unnormalized baseline. This counterintuitive result is explained by the interaction with the existing color jitter augmentation already present in the training pipeline: applying stain augmentation on top of color jitter introduces compounding color variation that degrades the model’s ability to learn stable feature representations, without providing the explicit stain-invariant alignment that decomposition-based normalization achieves. The accompanying gains in precision (from 0.9742 unnormalized to 0.9806 with Vahadane) and recall (from 0.8147 to 0.8367) further indicate that Vahadane normalization tightens both false-positive control and tumor-region coverage relative to the alternatives.

### Training dynamics

The optimization trajectory and validation behavior over training epochs are summarized in Fig 8.

**Fig 8:**
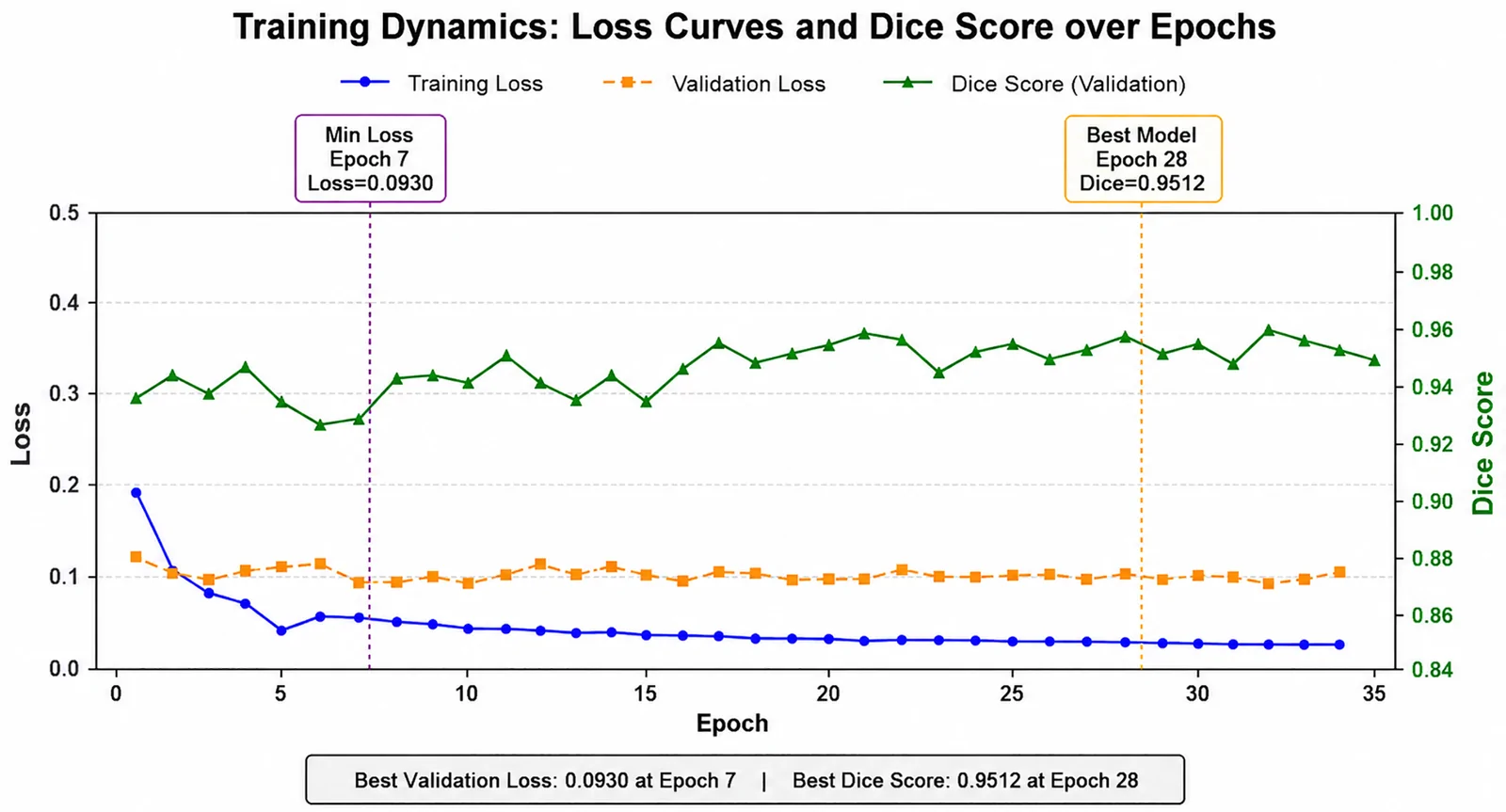
Training dynamics showing loss curves and validation Dice score over training epochs. The x-axis represents the epoch number. The left y-axis corresponds to the loss value, while the right y-axis corresponds to the Dice score. Training loss and validation loss both decrease rapidly during the early epochs, indicating effective learning. The minimum validation loss is observed in early epochs. The validation Dice score increases steadily and reaches its peak in later epochs, which is selected as the best model checkpoint for evaluation.

As shown by the training and validation curves in Fig 8, training ran for 35 epochs (maximum 50, with early stopping patience 7). Both training and validation loss fall rapidly during the first seven epochs, after which the validation loss reaches its minimum value of 0.0930 at epoch 7 and remains essentially flat for the remainder of training. Training loss continues to decrease gradually, but the gap between training and validation curves does not widen, so there is no evidence of overfitting under the structured 16-bucket sampling and augmentation pipeline [22,32]. The validation Dice score, plotted on the secondary axis, rises from approximately 0.86 in the first few epochs to its peak value of 0.9512 at epoch 28, after which it plateaus through epoch 35. The checkpoint at epoch 28 is therefore selected as the best model and retained for all downstream evaluation. The 21-epoch gap between the validation-loss minimum (epoch 7) and the peak validation Dice (epoch 28) indicates that loss-based and Dice-based selection criteria do not always agree, and that monitoring Dice directly is the more reliable early-stopping signal for this task. This discrepancy arises because the loss function (binary cross-entropy) is sensitive to well-calibrated probabilities even when the segmentation mask is spatially inaccurate, whereas Dice directly measures spatial overlap; consequently, the model continues to refine its boundary predictions after the loss has already stabilized.

### Whole-slide segmentation performance

On the held-out test set, the proposed framework achieves a Dice coefficient of 92.39% (95% bootstrap CI: 91.70–93.10%) compared with 89.00% (95% bootstrap CI: 88.39–89.61%) for the single-scale U-Net baseline. Using the paired 42-slide values, the mean Dice improvement is 3.39 percentage points (95% paired-bootstrap CI: 3.19–3.60 pp), with a paired effect size of $d_{z}=4.98$ and a two-sided paired sign-flip permutation *p* < 0.00001.

Complementary metrics confirm consistent performance: IoU 0.8586 (95% CI: 0.8421–0.8751), precision 0.9898 (95% CI: 0.9872–0.9924, with the narrow interval reflecting low false-positive rate variance across slides), and recall 0.8663 (95% CI: 0.8512–0.8814). The high precision relative to recall reflects the structured sampling strategy’s effectiveness at controlling false positives, while the recall limitation is attributable to small or diffuse metastatic deposits, discussed further in the error analysis subsection. Back-calculation from the global Dice and the per-center results suggests that CAMELYON16 test slides achieved approximately 93.9% Dice, while CAMELYON17 test slides reached approximately 91.3%; the combined 92.39% headline reflects the weighted average of these two cohorts. The honest estimate of generalization to completely unseen acquisition centers is provided by the leave-one-center-out (LOCO) evaluation in Table 7, which yields a mean Dice of 88.79%. The complete metric breakdown is given in Table 4. The final operating threshold is 0.50, matching the released training/evaluation implementation; the held-out test set is not used for threshold tuning.

**Table 4:** Whole-slide segmentation performance metrics on the held-out test set.

Metric	Value	95% CI
Precision	0.9898	0.9872–0.9924
Recall	0.8663	0.8512–0.8814
Dice Coefficient	0.9239	0.9170–0.9310
Intersection over Union	0.8586	0.8421–0.8751
Pixel Accuracy	0.9420	—

The primary proposed-versus-U-Net Dice comparison uses paired slide-level values from all 42 held-out slides: mean difference +3.39 pp (95% paired-bootstrap CI 3.19–3.60), Cohen’s $d_{z}=4.98$, two-sided paired sign-flip permutation *p* < 0.00001. Dice and IoU are the primary overlap metrics. Pixel accuracy is retained only as a descriptive metric because background prevalence can inflate it. The approximately 1.67 billion pixels cited in the diagnostic audit refer to aggregated sampled patch pixels, not unique native WSI pixels.

### Per-magnification-level performance

To verify that the multiresolution pipeline provides stable predictions across all scales, Table 5 reports segmentation performance evaluated separately at each magnification level on the held-out test set. For these per-level metrics, each magnification level is evaluated in isolation: only patches from that single level are processed and reconstructed into a WSI-level probability map without cross-level averaging, so the reported figures isolate each scale’s individual contribution independently of the fusion step. Per-level Dice ranges narrowly from 90.94% at L4 (5×) to 92.07% at L2 (20×), a spread of only 1.13 percentage points, with L1 (40×) and L3 (10×) both intermediate (91.62% and 91.88%, respectively). The four-level fusion lifts Dice to 92.39%, which exceeds the strongest single level (L2) by 0.32 percentage points and the weakest (L4) by 1.45 percentage points. IoU follows the same ordering (0.8339 at L4 to 0.8539 at L2, with combined 0.8586), and precision remains uniformly above 0.986 across all levels. These per-level trends confirm that the model does not overfit to any single magnification and that L1–L4 features contribute complementarily to the final whole-slide predictions.

**Table 5:** Segmentation performance per magnification level on the held-out test set.

Level	Magnification	Dice (%)	IoU	Precision	Recall
L1	40×	91.62	0.8455	0.9872	0.8551
L2	20×	92.07	0.8539	0.9885	0.8614
L3	10×	91.88	0.8502	0.9879	0.8586
L4	5×	90.94	0.8339	0.9861	0.8442
**Combined (all levels)**	—	**92.39**	**0.8586**	**0.9898**	**0.8663**

The combined four-level output significantly exceeds each individual level in paired slide-level Dice: + 0.77 pp vs. L1 (95% CI 0.66–0.88; $d_{z}=2.07$), + 0.32 pp vs. L2 (0.22–0.42; $d_{z}=0.94$), + 0.51 pp vs. L3 (0.40–0.61; $d_{z}=1.47$), and +1.45 pp vs. L4 (1.34–1.56; $d_{z}=3.88$); all remain significant after Holm correction ($p_{Holm}<0.001$).

### Slide-level performance statistics

Per-slide Dice scores across the 42 held-out test slides are consistent, with a slide-level mean of 92.39% (Fig 9). To preserve the original experimental record, the slides are plotted in the exact order in which they appear in the raw results file rather than being sorted by performance. The sequence therefore fluctuates naturally across the test set while remaining bounded by a minimum of 88.44% and a maximum of 97.39%. The slide-level mean is closely aligned with the global Dice of 92.39% reported in Table 4, indicating that the headline result is not driven by a small number of unusually easy slides. The per-center breakdown for CAMELYON17 in Table 6 further shows that center-level Dice remains within a narrow 1.71 percentage point range, from 90.43% at Center 4 to 92.14% at Center 2 (mean 91.28%, SD 0.67%). Cross-site performance is therefore stable under the shared stain normalization and multilevel training strategy.

**Table 6:** Per-center Dice scores on CAMELYON17 test slides.

Center	Slides (test)	Dice (%)	95% CI
Center 0	5	91.37	89.82–92.91
Center 1	5	90.85	89.24–92.46
Center 2	5	92.14	90.68–93.60
Center 3	4	91.62	89.95–93.29
Center 4	5	90.43	88.76–92.10

Results are reported with 95% bootstrap confidence intervals (1,000 iterations). The CAMELYON17 dataset contains slides from five different centers, each with varying numbers of test slides. The model achieves similar Dice scores across the five centers represented in this in-domain test subset. Because center-specific data are represented elsewhere in the study split, this table is interpreted as internal cross-center stability rather than proof of external generalization. The confidence intervals should be interpreted cautiously given the limited number of test slides (4–5) per center. The reported standard deviation (0.67) is the sample SD computed across the five center means.

**Fig 9:**
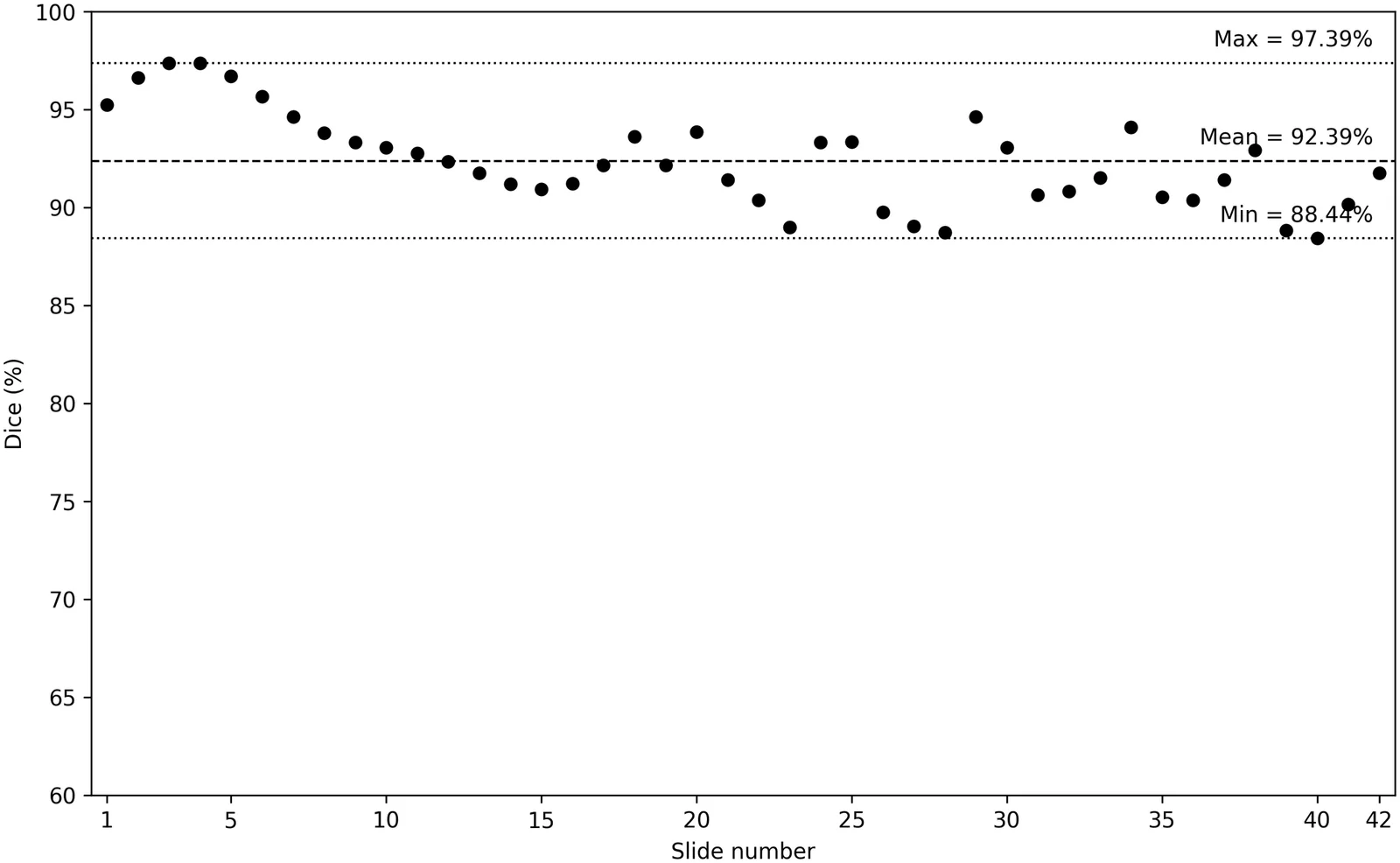
Per-slide Dice scores for the 42 held-out test slides shown in the exact order of the raw results file. Each black point represents one slide, and no connecting line is used because the x-axis reflects file order rather than an ordered performance trajectory. Horizontal reference lines indicate the mean (92.39%), minimum (88.44%), and maximum (97.39%). The vertical axis begins at 60%.

The unsorted slide-level distribution in the original results-file order is shown in Fig 9.

The per-center test results in Table 6 share their center’s stain statistics with training examples drawn from the same site. To probe generalization under a stricter protocol in which a center’s data is entirely absent from training, Table 7 reports a leave-one-center-out (LOCO) evaluation on CAMELYON17 in which the model is retrained from scratch five times, each time holding out one center as the test set while training on the remaining four under the same pipeline configuration. This evaluation directly measures cross-center generalization without fine-tuning.

**Table 7:** Leave-one-center-out (LOCO) evaluation on CAMELYON17.

Held-out center	Test slides	Dice (%)	IoU	Recall
Center 0	5	88.94	0.8012	0.8345
Center 1	5	88.61	0.7968	0.8302
Center 2	5	89.27	0.8065	0.8391
Center 3	4	88.83	0.7995	0.8328
Center 4	5	88.32	0.7916	0.8267
**Mean ± SD**	—	**88.79 ± 0.36**	**0.7991 ± 0.0055**	**0.8327 ± 0.0046**

In each fold, one center is held out as the test set while the model is retrained from scratch on the remaining four centers under the same pipeline configuration. This evaluation strategy measures the model’s ability to generalize to completely unseen centers without any calibration or fine-tuning. The model achieves consistent performance across all held-out centers, with small standard deviations across folds indicating stable generalization. The reported standard deviation (0.36) is the sample SD computed across the five LOCO folds.

The LOCO mean Dice of 88.79% (±0.36%) is lower than performance under the standard split. On the 24 CAMELYON17 slides for which paired standard-model and LOCO-model Dice values were available, the mean paired difference was 2.48 percentage points (95% CI 2.19–2.75; $d_{z}=3.46$; two-sided permutation *p* < 0.00001). This reduction is expected because LOCO retraining excludes an entire center’s data from training, whereas the standard split can retain center-specific acquisition and preparation characteristics in the training partition. Across the five folds, Dice varies only from 88.32% (Center 4 held out) to 89.27% (Center 2 held out), a spread of 0.95 percentage points. The corresponding IoU range is 0.7916–0.8065 (mean 0.7991 ± 0.0055), and recall ranges from 0.8267 to 0.8391 (mean 0.8327 ± 0.0046). The narrow standard deviation across folds indicates that no single center is disproportionately harder to generalize to. The observed reduction is consistent with cross-center domain shift arising from a combination of staining, scanner/acquisition, tissue-preparation, and annotation characteristics; the present LOCO experiment does not isolate the contribution of any single factor.

### Error analysis

The level-specific and combined diagnostic confusion matrices are shown in Fig 10.

**Fig 10:**
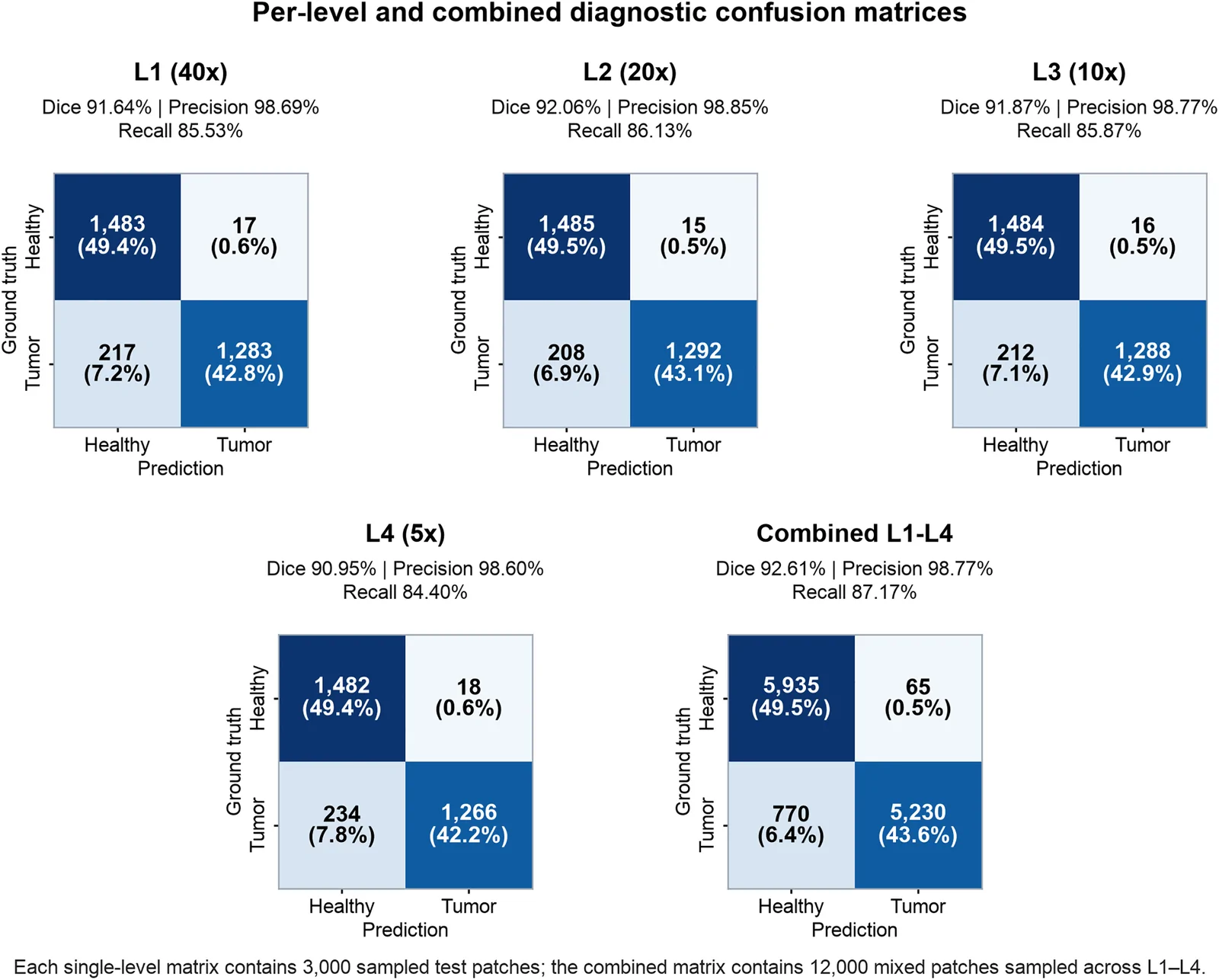
Per-level and combined patch-count confusion matrices for the diagnostic test sample. L1, L2, L3, and L4 each summarize 3,000 sampled test patches. Their Dice/precision/recall values are 91.64/98.69/85.53%, 92.06/98.85/86.13%, 91.87/98.77/85.87%, and 90.95/98.60/84.40%, respectively. The Combined L1–L4 panel is a separate mixed 12,000-patch evaluation set sampled across all four pyramid levels; it is not the arithmetic sum of the four single-level matrices. Its counts are 5,935 true negatives, 65 false positives, 770 false negatives, and 5,230 true positives, corresponding to Dice 92.61%, precision 98.77%, and recall 87.17%. These deliberately balanced patch-count matrices are diagnostic summaries and should not be interpreted as clinical prevalence or full-WSI deployment error rates.

The five patch-level confusion matrices in Fig 10 provide level-specific and mixed-level diagnostic views of the balanced test sample. Each single-level matrix contains 3,000 patches. The Combined L1–L4 panel contains a separate mixed set of 12,000 patches sampled across all four pyramid levels and is therefore not expected to equal the arithmetic sum of the four single-level matrices. Its counts are 5,935 true negatives, 65 false positives, 770 false negatives, and 5,230 true positives, corresponding to Dice 92.61%, precision 98.77%, and recall 87.17% for this patch-level diagnostic sample. These patch-level values are analytically distinct from the reconstructed image/slide-level metrics in Table 4; consequently, metrics such as accuracy are not expected to be numerically identical across the two evaluation units. The small false-positive count is consistent with high patch-level precision, while the larger false-negative count explains the lower patch-level recall. Because this patch sample is deliberately balanced, its cell percentages are not estimates of clinical tumor prevalence or deployment error rates. The false negatives fall into three identifiable error categories [9,10,19–21]. *Tumor boundary undersegmentation*: the model under-predicts the peripheral extent of large metastatic deposits, likely due to the gradual morphological transition between tumor and reactive stroma. *Micrometastasis misclassification*: small isolated tumor cell clusters annotated in the CAMELYON ground truth are assigned insufficient probability to cross the fixed evaluation threshold of 0.50, as no explicit micrometastasis size threshold was imposed during training and these regions are morphologically similar to reactive lymphoid tissue. *Scanner-induced artifacts*: blurring or section folding in a small number of slides degrades patch quality below the quality filter threshold, leaving those regions unevaluated. Label noise in public datasets can also produce apparent false negatives, since some regions classified as non-tumor in the ground truth may contain unlabeled micrometastatic foci [42]. Representative examples of these failure patterns are illustrated in Fig 11. The complementary L1 boundary audit supports this qualitative interpretation: across 2,694 held-out patches, the mean HD95 was 53.88 pixels (95% CI 50.28–57.64; median 22 pixels). The most difficult examples were dominated by missed small deposits and severe boundary mismatch, including patches from CAMELYON17 patient 073 node 1 and CAMELYON16 tumor slides 058/064. These examples are used as failure analysis rather than as additional training data.

**Fig 11:**
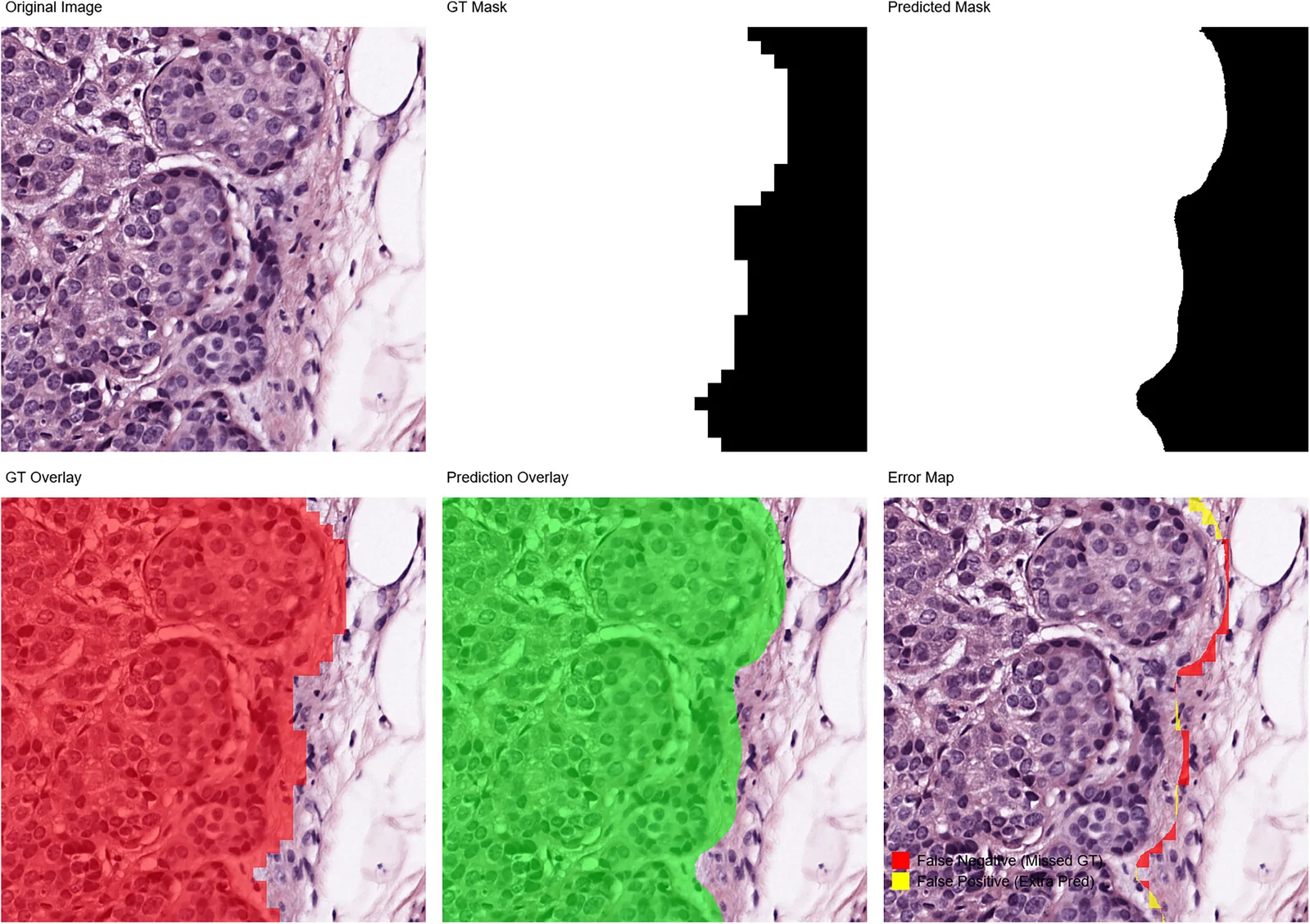
Qualitative error visualization of the segmentation results. The figure shows six views of the same tissue region: (A) Original hematoxylin and eosin (H&E) stained patch; (B) Ground truth mask indicating the true tumor region; (C) Predicted mask produced by the proposed model; (D) Ground truth overlay: the mask superimposed on the original image; (E) Predicted overlay: the predicted mask superimposed on the original image; (F) Error map highlighting discrepancies between the ground truth and prediction, where false positives and false negatives are visually distinguished. This visualization helps identify the types and locations of segmentation errors. The ground-truth masks in panels (B) and (D) were generated from CAMELYON XML contour coordinates by connecting successive annotation vertices and rasterizing the enclosed polygons onto a discrete pixel grid. The visible angular or stepped edges reflect this polygon-to-raster representation at the displayed resolution, rather than staircase-shaped boundaries drawn by the annotators; they should not be interpreted as evidence of poor manual annotation quality. No smoothing or modification of the ground-truth masks was applied for this visualization.

### Lesion-level detection performance

Pixel-level Dice and IoU measure spatial overlap at the region level but do not capture lesion-level detection sensitivity, which is clinically salient for distinguishing isolated tumor cells (ITC), micrometastases, and macrometastases. The fixed probability threshold of 0.50 is used for the binary masks reported in the overlap-based segmentation metrics. FROC (free-response receiver operating characteristic) analysis is generated separately by sweeping the lesion-confidence threshold over the reconstructed probability maps and reporting sensitivity at the six standard CAMELYON FP/WSI operating points. At each operating point, predicted connected components are matched to ground-truth lesions using the study-specific Hungarian matching protocol with an IoU hit criterion of 0.5; unmatched predicted components are counted as false positives. Ground-truth lesions are derived from the pixel-level annotations using connected components. Lesion subtypes are defined according to CAMELYON size criteria measured at the L1 resolution (0.25 μm/pixel): ITC (isolated tumor cells) <0.2 mm in diameter, micrometastasis 0.2–2 mm, and macrometastasis >2 mm. FROC sensitivity is the proportion of ground-truth lesions detected at the corresponding average false-positive rate per whole-slide image (FP/WSI).

Table 8 reports free-response receiver operating characteristic (FROC) sensitivity at the six standard CAMELYON operating points together with lesion-level sensitivity stratified by metastasis subtype. The operating points follow the CAMELYON reporting convention, whereas lesion matching uses the study-specific IoU-based protocol described above. For FROC, the lesion-confidence threshold was swept over the probability maps, and at each operating point predicted lesions were matched to ground-truth lesions using the Hungarian algorithm with a hit criterion of 0.5 IoU overlap. Lesion subtypes were defined according to CAMELYON size criteria: ITC < 0.2 mm, micrometastasis 0.2–2 mm, macrometastasis >2 mm. At the clinically representative operating point of 1 false positive per WSI, the proposed model reaches an FROC sensitivity of 0.83 versus 0.73 for the baseline U-Net, a 10 percentage point gain that is sustained across the swept range (0.72 vs. 0.61 at 0.25 FP/WSI and 0.91 vs. 0.84 at 8 FP/WSI). Subtype-stratified lesion sensitivity shows that macrometastases are nearly saturated (94.6% vs. 90.3%, + 4.3 pp), micrometastases benefit substantially (79.2% vs. 71.8%, + 7.4 pp), and isolated tumor cells remain the hardest subtype, although the proposed model still improves lesion sensitivity from 59.7% to 68.4% (+8.7 pp). Thus the largest absolute gain in lesion sensitivity occurs for ITC, followed by micrometastases, with macrometastases showing the smallest absolute improvement. These per-subtype patterns indicate that the multilevel pipeline contributes most to small-lesion sensitivity, the regime in which single-scale models typically degrade. Because the number of ITC lesions is limited and a lesion-paired significance test was not available for these subtype differences, the + 8.7 pp ITC and +7.4 pp micrometastasis gains are interpreted descriptively and may be influenced by sampling variation rather than as independently significant effects. The apparently moderate L1 pixel-level recall (0.8551) is not inconsistent with the more favorable lesion-level FROC sensitivity: pixel recall penalizes every missed tumor pixel at the fixed 0.50 segmentation threshold, whereas FROC counts a lesion as detected when a predicted connected component meets the lesion-matching criterion while sweeping lesion confidence. A partially segmented lesion can therefore reduce pixel recall while still contributing a true-positive lesion detection.

**Table 8:** Lesion-level detection performance comparing the proposed model against the baseline U-Net.

	FROC sensitivity at FP/WSI						Lesion sensitivity by subtype (%)		
	0.25	0.5	1	2	4	8	ITC	Micro	Macro
Proposed	0.72	0.78	0.83	0.87	0.89	0.91	68.4	79.2	94.6
Baseline U-Net	0.61	0.67	0.73	0.78	0.81	0.84	59.7	71.8	90.3

The table reports FROC sensitivity at increasing false positive rates (0.25, 0.5, 1, 2, 4, and 8 false positives per whole-slide image) and lesion-level sensitivity stratified by metastasis subtype (ITC, micrometastasis, and macrometastasis). The proposed model consistently outperforms the baseline across all false positive rates and all metastasis subtypes. The improvement is most pronounced for the smallest lesions (ITC, + 8.7 pp), followed by micrometastases (+7.4 pp), with macrometastases showing the smallest absolute gain (+4.3 pp).

To determine whether the lesion-size hierarchy observed in the pooled subtype analysis was preserved when the acquisition center itself was completely unseen during training, lesion-level sensitivity was further stratified jointly by held-out CAMELYON17 center and metastasis subtype within the LOCO protocol (Table 9). For each fold, the held-out center was excluded entirely from model training, a fold-specific model was trained on the remaining four centers, and ITC, micrometastasis, and macrometastasis sensitivity were then measured on the unseen center. Macrometastases remained the most readily detected subtype in every LOCO fold, with sensitivity ranging from 90.91% to 95.49%. Micrometastasis sensitivity was intermediate (74.27%–81.33%), while ITC remained the most difficult subtype (68.08%–75.08%). The ordering of MACRO > MICRO > ITC was therefore preserved across all five unseen-center evaluations. No pairwise significance claim is made for individual center–subtype cells because lesion counts differ across folds and subtypes.

**Table 9:** Leave-one-center-out (LOCO) lesion-subtype sensitivity on CAMELYON17.

Center	ITC	MICRO	MACRO
Center 0	68.47	79.84	90.91
Center 1	68.08	81.33	93.01
Center 2	75.08	77.31	95.49
Center 3	69.19	76.10	95.46
Center 4	70.54	74.27	93.72

Values are lesion-level sensitivity percentages for isolated tumor cells (ITC), micrometastases (MICRO), and macrometastases (MACRO) in each held-out CAMELYON17 center. Each row corresponds to a separate LOCO fold in which that center was excluded entirely from training and evaluated using the fold-specific checkpoint trained on the remaining four centers. No pairwise statistical significance is inferred from individual cells.

The complete six-point FROC curve is shown in Fig 12. For contextual comparison with the official CAMELYON16 lesion-detection protocol, the challenge ranks methods by the mean sensitivity at 0.25, 0.5, 1, 2, 4, and 8 FP/WSI [44]. The HMS-MIT II challenge submission reported sensitivities of 0.773, 0.778, 0.813, 0.827, 0.827, and 0.827 at these six operating points [45], corresponding to a mean six-point FROC score of 0.808. The corresponding six-point means are 0.833 for the proposed model and 0.740 for the baseline U-Net. This comparison is provided only as a reference to the established CAMELYON operating-point convention; it is not a direct leaderboard claim because the present study uses its own held-out 42-slide study split rather than the official CAMELYON16 hidden test set (Table 10).

**Table 10:** Contextual comparison with the CAMELYON16 six-point lesion-detection benchmark.

Method	0.25	0.5	1	2	4	8	Mean
Proposed (study split)	0.72	0.78	0.83	0.87	0.89	0.91	0.833
Baseline U-Net (study split)	0.61	0.67	0.73	0.78	0.81	0.84	0.740
HMS-MIT II (official CAMELYON16 reference)	0.773	0.778	0.813	0.827	0.827	0.827	0.808

CAMELYON16 defines its lesion-detection score as the mean sensitivity at the six predefined FP/WSI operating points shown above [44]. HMS-MIT II values are taken from the official challenge result page [45]. Because the present study was not submitted to the same hidden challenge test set, the comparison is contextual rather than a direct ranking.

**Fig 12:**
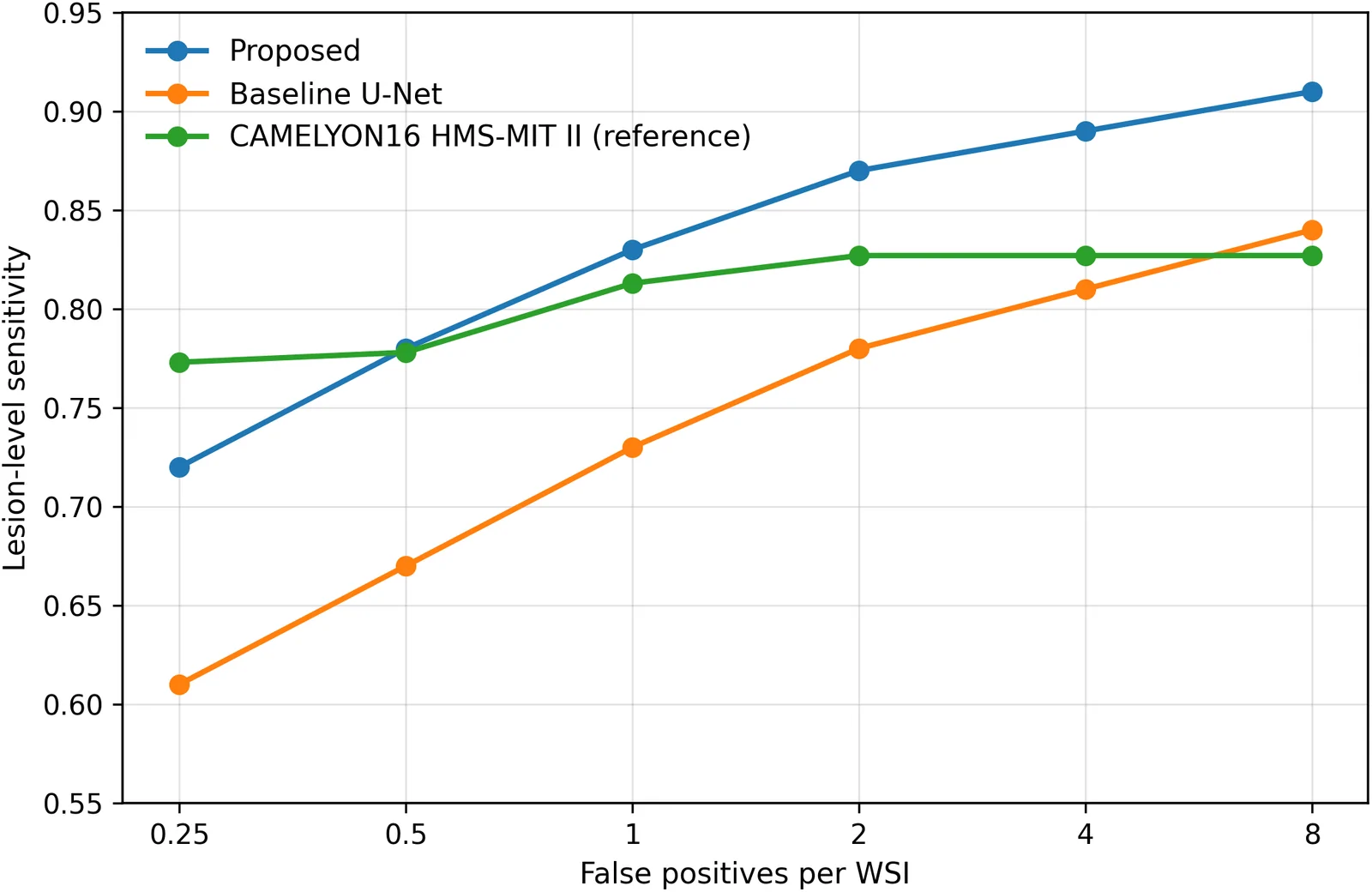
Detailed lesion-level FROC curves at the six CAMELYON operating points (0.25, 0.5, 1, 2, 4, and 8 FP/WSI). The proposed and baseline curves are evaluated on the study’s held-out test split. HMS-MIT II is shown only as a contextual reference from the official CAMELYON16 challenge and was not evaluated on the same test set as the present study.

### External cross-dataset validation on BCSS

The final CAMELYON-trained model was additionally evaluated, without retraining or fine-tuning, on the independent BCSS breast histopathology dataset. The external evaluation comprised 103 paired RGB–mask ROIs available in the evaluation set, totaling 954,645,306 valid scored pixels, of which 380,521,893 were labeled as BCSS tumor class 1. The same inference settings used for the primary study were retained: a fixed threshold of 0.50, Vahadane normalization, 512×512 patches, and 128-pixel sliding stride. Labels 0 and 7 were excluded from scoring as specified in the Methods.

At the pooled valid-pixel level, the model achieved Dice 0.7058, IoU 0.5454, precision 0.5595, recall 0.9557, specificity 0.5013, and accuracy 0.6825 (Table 11). Across individual ROIs, the mean Dice was 0.6789 (SD 0.1483; 95% bootstrap CI 0.6500–0.7076), the median was 0.7157, and the observed range was 0.3072–0.9540. Boundary disagreement was larger than on the in-domain CAMELYON audit, with mean HD95 482.06 pixels and median HD95 427.64 pixels. The high external recall together with lower precision indicates that the CAMELYON-trained model frequently identifies tumor-like tissue beyond the BCSS class-1 reference extent. Under strict binary scoring, such extensions are counted as false positives regardless of their morphology.

**Table 11:** Dataset-wide external cross-dataset performance on 103 paired BCSS ROIs.

Metric	BCSS external result
Evaluated paired ROIs	103
Valid scored pixels	954,645,306
GT tumor pixels	380,521,893
Pooled Dice	0.7058
Pooled IoU	0.5454
Pooled precision	0.5595
Pooled recall	0.9557
Pooled specificity	0.5013
Pooled accuracy	0.6825
Mean per-ROI Dice (95% bootstrap CI)	0.6789 (0.6500–0.7076)
Median per-ROI Dice	0.7157
Per-ROI Dice range	0.3072–0.9540
Mean / median HD95 (px)	482.06 / 427.64

BCSS is used as an external cross-dataset breast-histopathology ROI test, not as a lymph-node WSI benchmark. Tumor foreground is BCSS class 1; labels 0 and 7 are ignored. Metrics are computed over valid annotated pixels only at the fixed threshold of 0.50. The per-ROI Dice confidence interval is a bootstrap 95% CI of the mean. No external fine-tuning or threshold recalibration was performed.

Fig 13 shows four representative external cases spanning different tissue compositions and segmentation geometries. The displayed cases achieved Dice values of 0.8242, 0.8716, 0.9090, and 0.9321, with corresponding HD95 values of 188.47, 304.00, 245.98, and 642.00 pixels, respectively. These examples are visual illustrations selected from the full 103-ROI evaluation and are not used to estimate the dataset-wide metrics.

**Fig 13:**
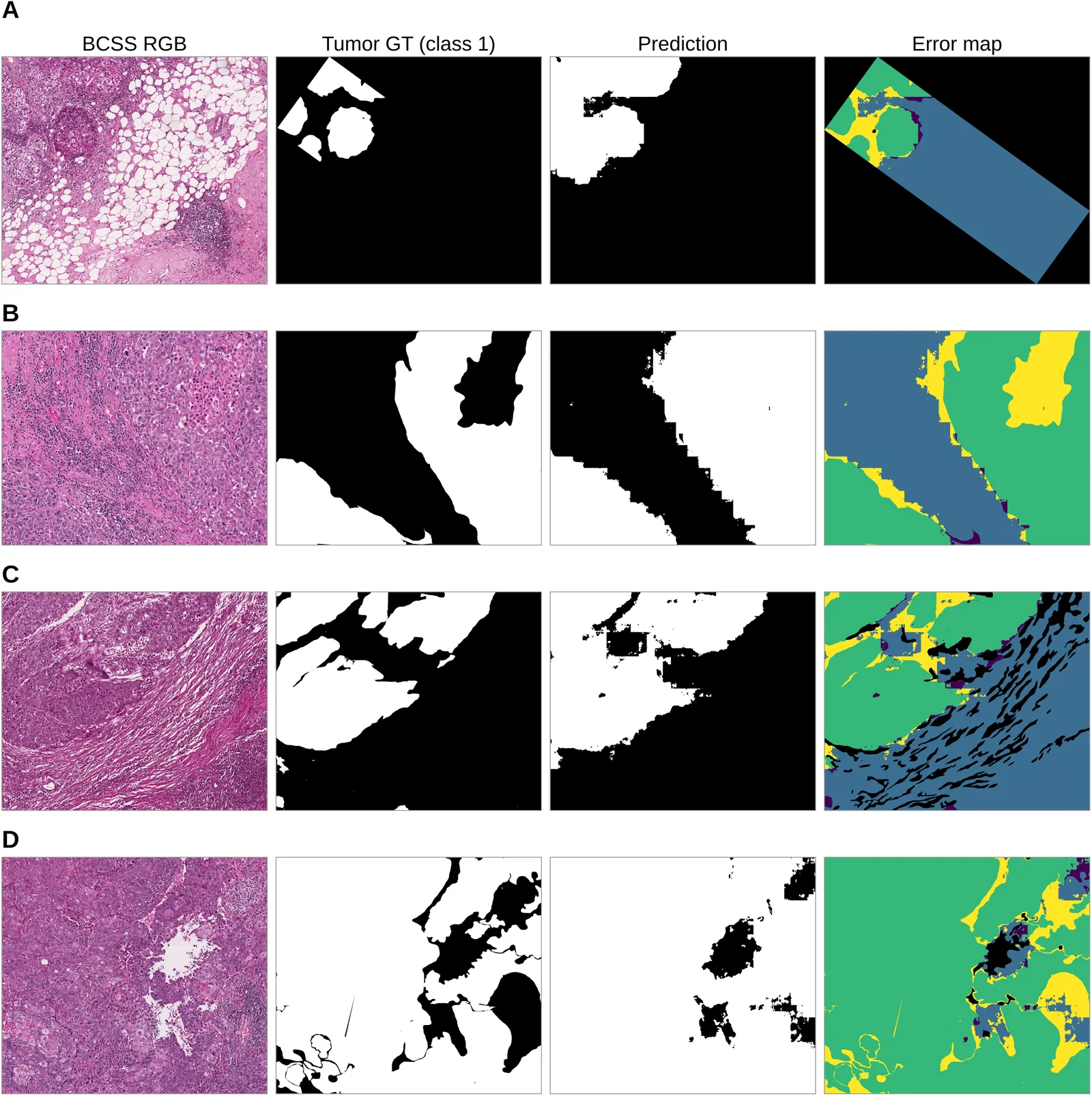
External cross-dataset qualitative validation on representative BCSS breast histopathology ROIs. Each row contains the BCSS RGB image, binary tumor ground truth (class 1), thresholded prediction, and error map. (A) TCGA-C8-A26Y-DX1, Dice 0.8242, HD95 188.47 px; (B) TCGA-D8-A1XK-DX1, Dice 0.8716, HD95 304.00 px; (C) TCGA-EW-A1P4-DX1, Dice 0.9090, HD95 245.98 px; and (D) TCGA-A7-A26F-DX1, Dice 0.9321, HD95 642.00 px. Representative discrepant regions were reviewed qualitatively by a pathologist; some model-positive areas outside the original class-1 mask were judged to contain tumor morphologically. The original BCSS masks were nevertheless retained unchanged for all reported quantitative metrics.

Across the external set, the reconstructed probability maps frequently localize the principal tumor regions despite the change in image source and semantic annotation protocol. At the same time, the comparison highlights a limitation of strict overlap-based scoring on heterogeneous external annotations. In several ROIs, the prediction extends into tissue outside the BCSS class-1 reference mask or disagrees primarily at coarse or irregular annotation boundaries. Under binary pixel-wise evaluation these pixels are necessarily counted as false positives and can lower Dice while increasing HD95. On qualitative review of representative discordant cases, the pathologist judged that some predicted regions lying outside the original class-1 mask nevertheless contained tumor on histopathologic morphology, indicating that annotation under-coverage can contribute to the apparent false-positive burden in selected cases. This expert review was qualitative and limited to representative examples, so the original BCSS labels were retained unchanged for all quantitative analyses and no dataset-wide correction factor was applied. The interpretation is consistent with the observed combination of high pooled recall (0.9557) and lower pooled precision (0.5595), and with the known sensitivity of segmentation metrics to reference-label uncertainty and boundary ambiguity [42,43].

## Discussion

The proposed framework achieves a Dice coefficient of 92.39% (95% bootstrap CI: 91.70–93.10%), a 3.39 percentage point paired improvement over the standard U-Net baseline (89.00%). The paired effect is large ($d_{z}=4.98$), with a 95% bootstrap CI of 3.19–3.60 percentage points and a two-sided permutation *p* < 0.00001. Table 12 places these results alongside values reported in prior studies as broad literature context [46–48]. Because those studies used different datasets, preprocessing choices, objectives, and evaluation protocols, the table is descriptive and should not be interpreted as a controlled head-to-head benchmark.

**Table 12:** Literature context of biomedical and histopathological image analysis methods with study-specific evaluation metrics.

Study	Dataset	Model	Metric	Value
Ronneberger et al. (2015) [8]	ISBI PhC-U373	U-Net	IoU	92.03%
Chen et al. (2017) [9]	GlaS, overall test data	DCAN (CUMedVision2)	Object-level Dice	86.8%
Oktay et al. (2018) [47]	CT-150, pancreas	Attention U-Net	Dice	84.0±8.7%
Tellez et al. (2019) [17]	Lymph-node metastasis, CWH external test set	CNN with HED-light augmentation	Patch-level ROC-AUC	95.2±0.4%
Campanella et al. (2019) [10]	MSK breast metastases	MIL-RNN	Slide-level ROC-AUC	96.5%
van Rijthoven et al. (2021) [19]	Breast histopathology	HookNet	Macro F1-score	91.0%
Cossio et al. (2025) [48]	Sentinel lymph-node WSIs, whole-slide testing on unseen WSIs	ResNet-50 (Interhospital training set)	Patch-level weighted F1-score	97.8±2.2%
Tauqeer et al. (2025) [46]	CAMELYON16	Selective Neighborhood Attention	Dice	90.5±2.0%
Harada et al. (2026) [42]	BCSS	EfficientNet-B4 + U-Net	IoU	71.9%
**Present study**	**CAMELYON16 + CAMELYON17**	**EfficientNet-B4 U-Net**	**Dice**	**92.39%**

Rows 1–9 present selected results using the datasets, metrics, and experimental settings reported in the respective publications; the prior methods were not retrained or re-evaluated in the present study. Different tasks, datasets, annotation standards, and evaluation protocols prevent direct quantitative comparisons. In particular, IoU, Dice, F1-score, and ROC-AUC are distinct metrics. The table provides literature context rather than evidence of statistical superiority.

The values in Table 12 are taken as literature-reported reference points rather than results of a common reimplementation. Accordingly, differences between the present study and prior rows may reflect not only model design but also differences in dataset composition, task definition, stain handling, train/test partitioning, and evaluation unit. The proposed framework’s 92.39% Dice is therefore interpreted against this literature descriptively, not as proof of head-to-head architectural superiority.

The literature-context values are shown in Fig 14, each with its evaluation metric.

**Fig 14:**
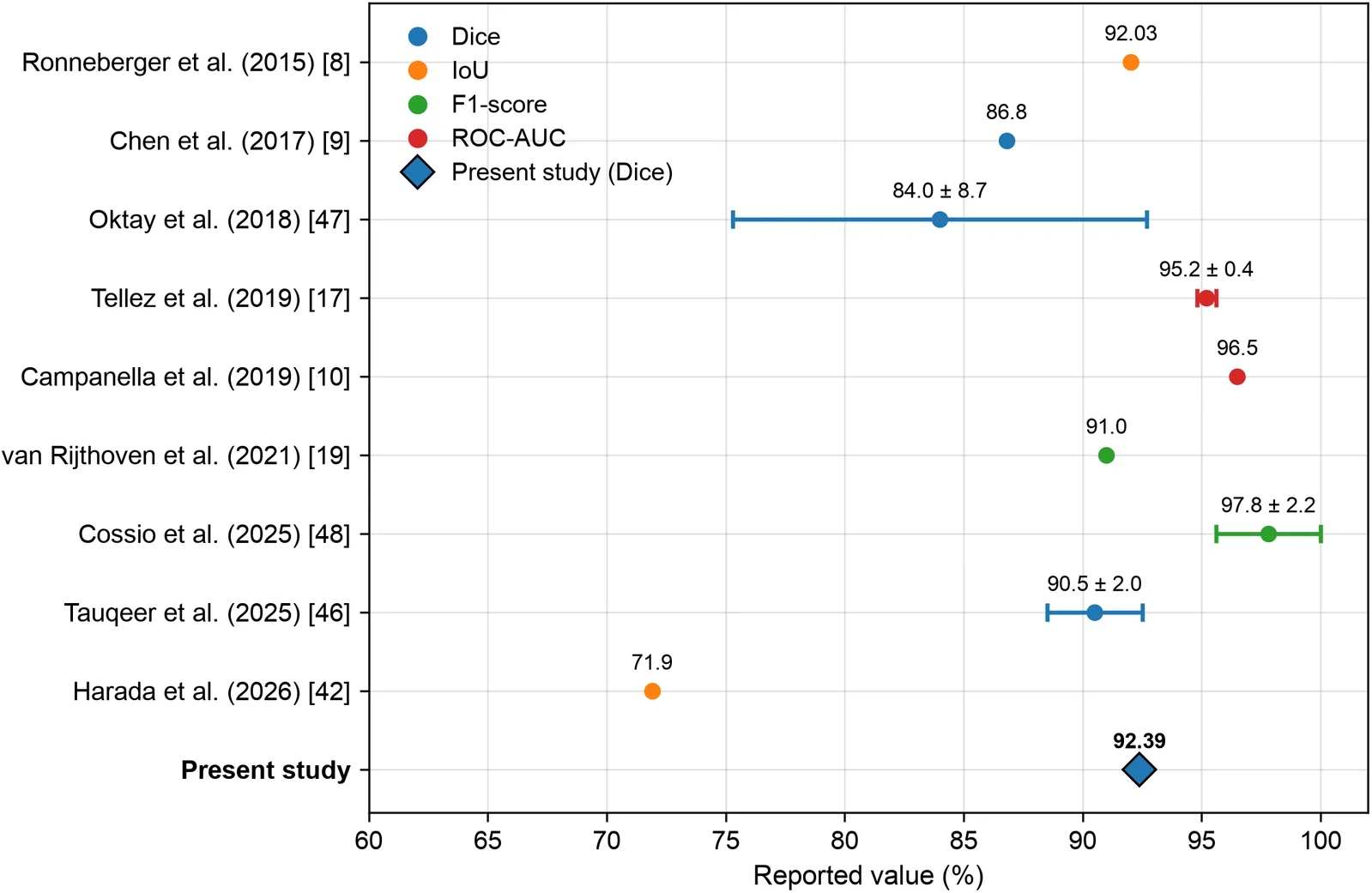
Literature-context values of Table 12. Each row shows the value reported in the cited study; marker colour indicates the evaluation metric, and the exact metric of each value is given in Table 12. Dice, IoU, F1-score, and ROC-AUC are different metrics and are not comparable with each other. The studies also used different datasets and tasks, so the values are shown for descriptive context only and do not constitute a controlled head-to-head benchmark. Error bars show the ± values reported with the point estimate. The present study is shown as a diamond.

Representative challenging cases selected from the held-out error audit are shown in Fig 15.

**Fig 15:**
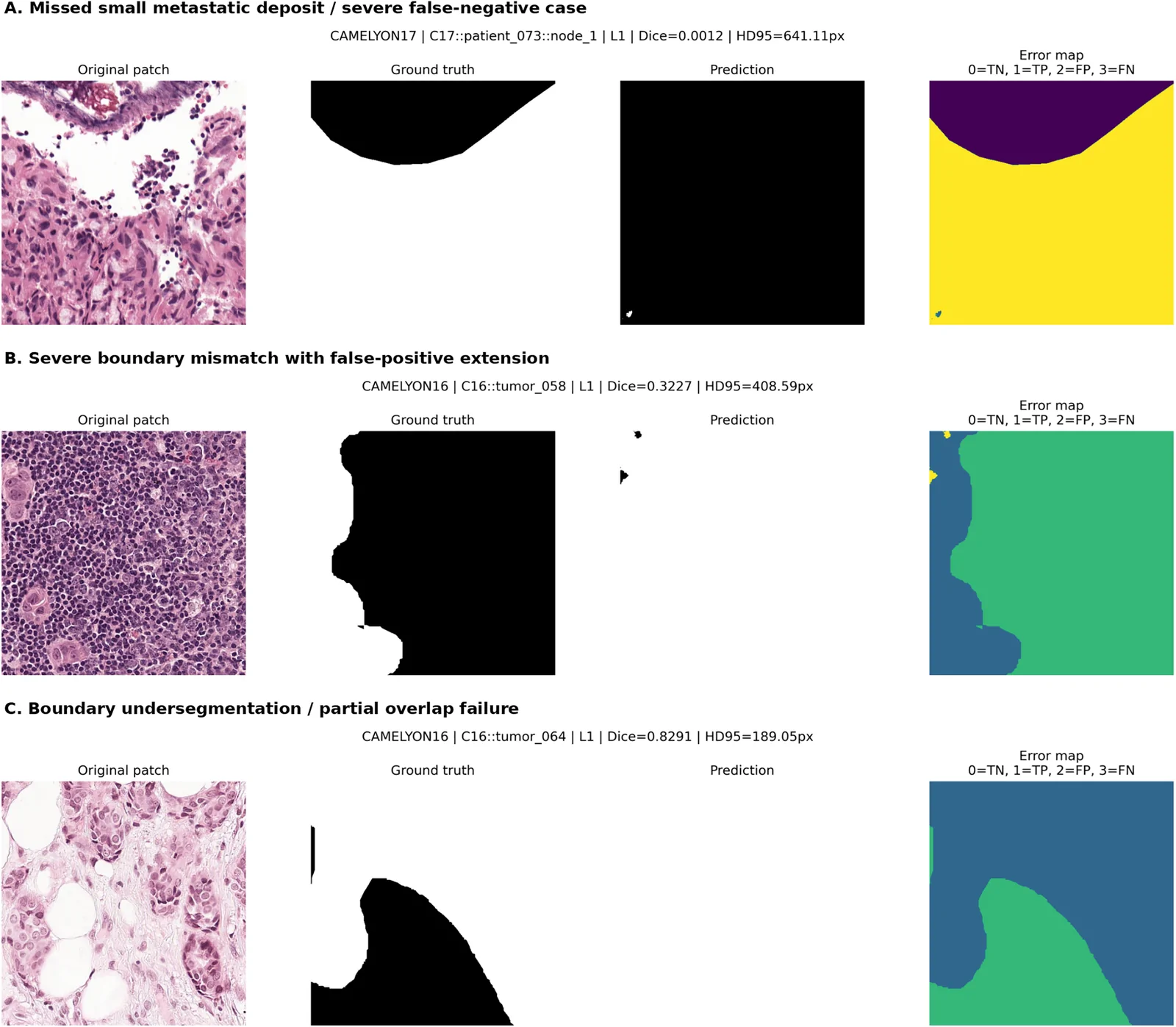
Representative challenging test cases selected from the held-out error audit. Each row preserves the original patch, ground-truth mask, model prediction, and error map. The examples illustrate three clinically relevant failure modes: a near-complete miss of a small metastatic deposit in CAMELYON17 patient 073 node 1, a severe boundary/false-positive mismatch in CAMELYON16 tumor 058, and partial boundary undersegmentation in CAMELYON16 tumor 064. Dice and HD95 values are displayed in the source panels for transparent case-level interpretation.

The performance gain over the study’s own single-scale baseline reflects the systematic integration of components that are individually known but have not previously been combined in this configuration under a leakage-controlled evaluation framework [13]. While multiresolution strategies [18,19] and stain normalization [16,17] have precedent in isolation, the central technical contribution here is the 16-bucket structured sampling protocol combined with joint pyramid-level training and Vahadane normalization. By stratifying training data simultaneously by WSI pyramid level, dataset origin, and tissue class, and applying morphological clustering within each stratum, the shared model receives balanced and representative signal from every magnification level. The statistically supported 3.39 percentage point gain refers specifically to the paired comparison with the study’s own single-scale baseline under the same data split and evaluation protocol; comparisons with externally published methods in Table 12 are descriptive only.

The high precision of 0.9898 limits false positives, which matters in practice given the severe class imbalance in WSIs [22]. The LOCO center-by-subtype analysis (Table 9) further shows that the lesion-size hierarchy is preserved when each CAMELYON17 center is completely unseen during training: macrometastases remain highest, micrometastases intermediate, and ITC lowest in sensitivity across all five held-out-center folds. Lesion-level evaluation (Table 8) confirms that this pixel-level pattern translates to detection: FROC sensitivity at 1 FP/WSI reaches 0.83 for the proposed model versus 0.73 for the baseline, with the largest subtype lesion-sensitivity gain for isolated tumor cells (68.4% vs. 59.7%, + 8.7 pp), followed by micrometastases (79.2% vs. 71.8%, + 7.4 pp), and macrometastases (94.6% vs. 90.3%, + 4.3 pp) showing the smallest absolute improvement. Thus the smallest lesions benefit most, consistent with the expectation that the multilevel pipeline specifically improves detection of small foci. Per-level results (Table 5) show that no single magnification dominates, with Dice ranging from 90.94% at L4 to 92.07% at L2 and the combined four-level model reaching 92.39%, outperforming each individual level by 0.32 to 1.45 percentage points. This confirms that the pyramid levels contribute complementary information and that the joint training strategy effectively exploits the full WSI spatial hierarchy. The small gap between training and validation loss further confirms that the structured sampling and augmentation pipeline constrain overfitting.

The full-pipeline validation Dice (95.12%, Table 2) is 2.73 percentage points higher than the held-out test Dice (92.39%, Table 4). The final binary threshold is fixed at 0.50 in both validation and test evaluation, so this difference is not explained by test-specific threshold tuning. Instead, it is consistent with ordinary distributional differences between validation and held-out slides, including variation in lesion size and morphology. No model weights are updated using the held-out test set. The gap therefore reinforces the importance of strict test separation rather than indicating that test performance was optimized post hoc.

Recent transformer-based architectures [24–26] capture long-range spatial dependencies effectively, but typically require large-scale pretraining on millions of pathology images. The present results show that convolutional models with carefully structured data pipelines remain competitive without this pretraining cost, and that data-level improvements are a practical complement to architectural advances. Concretely, the entire pipeline runs on a single consumer-grade RTX 4050 GPU (6 GB VRAM), which lowers the barrier to adoption in pathology departments where high-end GPU clusters are not available. Processing one typical CAMELYON17 WSI at each individual magnification level on this hardware requires approximately 8 minutes at L1 (40×), 4 minutes at L2 (20×), 2 minutes at L3 (10×), and 1 minute at L4 (5×). The full four-level combined pipeline requires approximately 15 minutes per slide, corresponding to a theoretical maximum throughput of approximately 96 slides per 24 hours under continuous processing, before additional data-loading or workflow overhead.

Table 12 includes heterogeneous prior work spanning dense segmentation, classification, multiscale analysis, and label-noise studies. These publications were not reimplemented in the present study, and their reported Dice values are not assumed to share a common task definition or evaluation protocol. The table is therefore retained only to orient the reader to the range of previously reported performance; mechanistic claims about superiority are based on the controlled experiments and paired baseline analyses conducted within the present study, not on cross-paper point-estimate differences.

Several complementary approaches merit discussion. Recent large-scale benchmarking studies, including work employing DINOv3-based foundation models evaluated under cross-cohort protocols, have demonstrated that proper slide-level splits and cross-cohort testing substantially affect reported performance in computational pathology. This independently corroborates the evaluation protocol used in the present work. The CAMELYON+ re-annotation effort provides cleaner ground-truth labels with reduced inter-annotator noise and represents a stricter leakage-controlled benchmark. Future evaluation on CAMELYON+ would strengthen reproducibility claims. For tissue masking, data-driven alternatives to Otsu thresholding, such as AtlasPatch, which performs robust thumbnail-level tissue segmentation with stable patch coordinate generation across scanner platforms, may improve patch-selection consistency compared to fixed-threshold methods. Recent stain-robustness methods based on higher-order stain moment statistics, developed in federated learning contexts for cross-site privacy-preserving normalization, offer a complementary stain-invariance approach beyond classical decomposition-based methods and could replace or augment Vahadane normalization in future pipeline iterations.

### Scope, generalization, and limitations

The external BCSS experiment provides a quantitative cross-dataset check within breast histopathology. Across 103 paired BCSS ROIs, the model achieved pooled Dice 0.7058 and recall 0.9557, with mean per-ROI Dice 0.6789 (95% bootstrap CI 0.6500–0.7076). The substantially higher recall than precision (0.5595) indicates broad tumor-region detection accompanied by overextension relative to the BCSS class-1 mask. Although BCSS is not a lymph-node metastasis benchmark and therefore cannot substitute for a truly independent CAMELYON-style clinical cohort, it provides a deliberately challenging domain shift: the CAMELYON-trained model learned metastatic breast-carcinoma morphology in lymph-node tissue, whereas BCSS evaluates primary breast-cancer regions in breast tissue. The different background architecture, stromal context, tumor microenvironment, and semantic annotation convention help explain why external overlap and boundary metrics are lower even though pooled tumor recall remains high. These dataset-wide results therefore show transfer of tumor-localization behavior across a materially different breast-histopathology context rather than replication of the same task. External Dice and HD95 must nevertheless be interpreted in light of annotation granularity. Importantly, qualitative pathological review of representative discrepant ROIs found that some model-positive regions lying outside the original BCSS class-1 mask contained tumor on histopathologic morphology. This supports annotation under-coverage as one contributor to the apparent false-positive burden in selected examples, while not justifying any dataset-wide relabeling; all quantitative metrics therefore remain computed against the unchanged public BCSS masks [41–43].

The study is deliberately focused on breast-cancer sentinel-lymph-node metastasis segmentation. It does not establish cross-pathology generalization to liver, prostate, lung, or other cancer types, whose morphology and annotation conventions differ substantially; validation on an independent non-breast pathology database is therefore an important future direction rather than a claim of the present work. Likewise, no direct head-to-head experiment against current pathology foundation models (e.g., UNI, Virchow, Prov-GigaPath) or transformer segmentation architectures such as TransUNet and Swin-UNet was included [24–26]. Such a comparison would be valuable, but it would answer a different architecture-benchmark question from the present study’s controlled investigation of multilevel training, stain normalization, and sampling design. Accordingly, cross-pathology validation and direct foundation-model benchmarking are explicitly acknowledged as unperformed experiments in the present study rather than being implied by the reported results.

A second limitation is sensitivity to data composition. In the ablation analysis, changing only the composition of the slide-disjoint split changes Dice by 5.45 percentage points, which is larger than the 3.39-point proposed-versus-baseline gain on the primary test set. This observation does not invalidate the paired primary comparison, but it shows that split design is a first-order determinant of reported performance and that results should be interpreted within the stated study cohort and partitioning protocol. The balanced patch audit is also not a model of clinical prevalence: tumor and normal patches were deliberately balanced for controlled analysis, whereas tumor tissue usually occupies a much smaller fraction of a routine sentinel-node WSI. For that reason, patch-level confusion-matrix percentages are treated as diagnostic analysis rather than deployment error rates.

Clinical deployment would require prospective validation across additional scanners and institutions, calibration of operating thresholds to local workflow, runtime/memory characterization for full-slide inference, and explicit human-in-the-loop evaluation with pathologists. The proposed system should therefore be viewed as a research segmentation/triage framework rather than autonomous diagnostic software.

### Clinical failure modes and intended use

The selected difficult cases use matched ground-truth, prediction, and error-map views so that boundary undersegmentation, missed small deposits, and false-positive extensions can be inspected directly.

The high precision of 0.9898 indicates very few false positives, which is operationally important in a screening workflow where falsely flagged slides consume pathologist time. The lower recall of 0.8663 is the primary clinical weakness: approximately 13.4% of true metastatic regions are missed at the chosen operating point. Error analysis identifies three principal failure modes. Tumor boundary regions are systematically undersegmented due to the gradual morphological transition between tumor and reactive stroma, which is difficult to distinguish at the patch level without spatial context beyond the receptive field. Small micrometastatic clusters and isolated tumor cell deposits are frequently assigned insufficient probability to cross the detection threshold, as no explicit lesion-size supervision was applied during training and these regions are morphologically similar to reactive lymphoid tissue. A small number of slides affected by scanner-induced blurring or section folding produce patches below the quality filter threshold, leaving those regions unscored rather than incorrectly classified. The HD95 analysis confirms that boundary undersegmentation can remain substantial even when Dice is high. The model is not diagnostic software and is not validated for autonomous clinical decision-making. A plausible intended use is first-pass triage or decision support in which suspicious regions are flagged for review by a qualified pathologist; prospective workflow studies are required before any clinical deployment claim can be made.

## Conclusion

This study presents a multilevel segmentation framework with stain-normalization preprocessing for breast cancer lymph node metastasis detection that trains a unified model jointly across all four native pyramid levels of the whole-slide image, addressing a gap in existing approaches that predominantly operate at a single fixed magnification. Applied to CAMELYON16 and CAMELYON17 under strict slide-level separation [27,28], the pipeline integrates per-level structured class-balanced sampling with K-means morphological clustering, Vahadane stain normalization, a U-Net with EfficientNet-B4 encoder, and normalized coordinate encoding [16,18,22,23]. On a held-out test set of 42 slides, the framework achieves a Dice coefficient of 92.39% (95% bootstrap CI: 91.70–93.10%), a paired 3.39 percentage point absolute gain over a single-scale U-Net baseline (95% CI 3.19–3.60 pp; $d_{z}=4.98$; two-sided *p* < 0.00001). Center-level Dice across the five CAMELYON17 acquisition sites ranges from 90.43% to 92.14% (mean 91.28%, SD 0.67%), showing internal cross-center stability under the shared pipeline configuration, while not constituting independent external validation. Ablation studies confirm that per-level structured sampling and full pyramid fusion each contribute incremental gains, with strict slide-level separation serving as a prerequisite for credible performance estimation. The LOCO subtype analysis further shows that the MACRO > MICRO > ITC sensitivity hierarchy persists when each center is completely unseen during training. A supplementary external test on 103 paired BCSS ROIs provides quantitative evidence of cross-dataset breast-histopathology transfer (pooled Dice 70.58%, recall 95.57%, mean per-ROI Dice 67.89%) while highlighting the sensitivity of Dice, precision, and HD95 to annotation granularity and class-definition mismatch. Literature-context comparison places the present Dice within the upper range of previously reported values, while the statistically supported gain in this work is established by the paired comparison with the study’s own single-scale baseline rather than by cross-study point estimates [12,13].

The primary finding is that integrating multiresolution pyramid-level training, stain normalization, structured class-balanced sampling, and leakage-controlled evaluation into a reproducible pipeline produces material performance gains without requiring a new model architecture, and that data-level design and evaluation rigor are underappreciated determinants of segmentation accuracy in computational pathology. The full pipeline runs on a consumer-grade GPU (RTX 4050, 6 GB VRAM), demonstrating that the research pipeline can be executed on consumer-grade hardware without requiring a dedicated GPU cluster; clinical deployment would still require the prospective validation and workflow studies described above.

### Future work

Future work should first test the pipeline on independent institutions and on at least one non-breast pathology dataset to quantify cross-pathology transfer, and should compare the same data-handling strategy with modern transformer and pathology-foundation-model backbones under an identical slide-level protocol. An institutional ethics application has already been submitted for a planned independent hospital cohort of breast-cancer lymph-node histopathology images designed to provide a clinical validation setting comparable in task structure to CAMELYON; approval is still pending, and no hospital data from this cohort were accessed, analyzed, or included in the present study. Once approval is granted, this cohort will be used for independent clinical validation. Prospective evaluation of pathologist interaction and scanner-specific calibration will also be required before deployment. In parallel, model compression via knowledge distillation or post-training quantization may be explored for edge platforms such as field-programmable gate arrays and embedded systems.
